# Five omic technologies are concordant in differentiating the biochemical characteristics of the berries of five grapevine (*Vitis vinifera* L.) cultivars

**DOI:** 10.1186/s12864-015-2115-y

**Published:** 2015-11-16

**Authors:** Ryan Ghan, Steven C. Van Sluyter, Uri Hochberg, Asfaw Degu, Daniel W. Hopper, Richard L. Tillet, Karen A. Schlauch, Paul A. Haynes, Aaron Fait, Grant R. Cramer

**Affiliations:** Department of Biochemistry and Molecular Biology, University of Nevada, Reno, Reno, NV 89557 USA; Department of Biological Sciences, Macquarie University, North Ryde, NSW 2109 Australia; Ben-Gurion University of the Negev, Jacob Blaustein Institutes for Desert Research, Midreshet Ben-Gurion, 84990 Israel; Nevada Center for Bioinformatics, University of Nevada, Reno, Reno, NV 89557 USA; Department of Chemistry and Biomolecular Sciences, Macquarie University, North Ryde, NSW 2109 Australia

**Keywords:** Grape berry, High-throughput sequencing, Metabolomics, Proteomics, Transcriptomics, *Vitis vinifera* L., Water-deficit stress

## Abstract

**Background:**

Grape cultivars and wines are distinguishable by their color, flavor and aroma profiles. Omic analyses (transcripts, proteins and metabolites) are powerful tools for assessing biochemical differences in biological systems.

**Results:**

Berry skins of red- (Cabernet Sauvignon, Merlot, Pinot Noir) and white-skinned (Chardonnay, Semillon) wine grapes were harvested near optimum maturity (°Brix-to-titratable acidity ratio) from the same experimental vineyard. The cultivars were exposed to a mild, seasonal water-deficit treatment from fruit set until harvest in 2011. Identical sample aliquots were analyzed for transcripts by grapevine whole-genome oligonucleotide microarray and RNAseq technologies, proteins by nano-liquid chromatography-mass spectroscopy, and metabolites by gas chromatography-mass spectroscopy and liquid chromatography-mass spectroscopy. Principal components analysis of each of five Omic technologies showed similar results across cultivars in all Omic datasets. Comparison of the processed data of genes mapped in RNAseq and microarray data revealed a strong Pearson’s correlation (0.80). The exclusion of probesets associated with genes with potential for cross-hybridization on the microarray improved the correlation to 0.93. The overall concordance of protein with transcript data was low with a Pearson’s correlation of 0.27 and 0.24 for the RNAseq and microarray data, respectively. Integration of metabolite with protein and transcript data produced an expected model of phenylpropanoid biosynthesis, which distinguished red from white grapes, yet provided detail of individual cultivar differences. The mild water deficit treatment did not significantly alter the abundance of proteins or metabolites measured in the five cultivars, but did have a small effect on gene expression.

**Conclusions:**

The five Omic technologies were consistent in distinguishing cultivar variation. There was high concordance between transcriptomic technologies, but generally protein abundance did not correlate well with transcript abundance. The integration of multiple high-throughput Omic datasets revealed complex biochemical variation amongst five cultivars of an ancient and economically important crop species.

**Electronic supplementary material:**

The online version of this article (doi:10.1186/s12864-015-2115-y) contains supplementary material, which is available to authorized users.

## Background

Grapes (*Vitis vinifera* L.) are an economically important agricultural commodity, having an economic impact greater than $162 billion to the American wine and grape industry alone (http://www.ngwi.org). Cultivated grapes are grown and consumed as fresh fruit, used as the root stocks for fruit producing scions, and in the production of a range of wines with distinct and complex flavor profiles [1]. Grapevines are a long-lived perennial fruit species intertwined within the culture of many countries dating back more than 7000 years.

There are more than 5000 distinct cultivars of grapes in the world. Grape production is found on every arable continent around the globe [2, 3]. Grapevines have maintained a rich genetic diversity since domestication as a result of vegetative propagation practices that both immortalize existing traits and unknowingly encourage unique phenotypes to arise from clonal cuttings that carry somatic mutations [2, 4]. Regional environments often referred to as “terroir”, in conjunction with human selective pressures have shaped the cultivar characteristics associated with many of the popular wines enjoyed today [5].

The color of a grape berry’s skin contributes a recognizable cultivar characteristic that differentiates red- and white-skinned grapes. Anthocyanins are the purple, blue and red pigments that provide the color associated with the skins and wines from red cultivars, and are extracted from the berry skins during winemaking; they are crucial constituents for quality in high-end wines [6]. White cultivars do not synthesize anthocyanins as a result of two adjacent mutations within the genes of the MYB transcription factors, in *VviMYBA1* and *VviMYBA2* [7, 8]. Human selective pressures from domestication are believed to have maintained this phenotype in many of today’s popular cultivars [2].

Other phenylpropanoids, besides anthocyanins, contribute to distinct cultivar differences in both grapes and wine. For example, genetic and environmental factors account for cultivar-dependent differences in abundance of the flavon-3-ols, catechin and epicatechin, in red wines produced from diverse regions [9]. Wine and table grapes also differ in their concentrations of both hydroxybenzoic and hydroxycinnamic acids levels, with wine grape content significantly higher [10]. The qualities of bitterness and astringency in wine are attributed to monomeric flavan-3-ols and polymeric proanthocyanidins or condensed tannins [11–14], and have been studied for their effects upon human health, including antioxidant and anti-inflammatory properties [15–18].

Cultivar differences also extend to subtle variations in amino acid composition at harvest [19–21]. Ammonia and certain amino acids are the main nitrogen-containing compounds assimilated by yeasts within fresh grape juice or musts before fermentation commences [22]. Nitrogenous substances become available to yeasts from pressed berry juice or via extraction from the skins, in the case of fermenting red wines. The assimilable nitrogen levels in grapes must also play a role in determining the duration of fermentation, and musts are often amended with ammonium salts (DAP) to ensure efficient fermentation [22]. Yeast assimilates free amino acids under anaerobic fermentation conditions, with the exception of proline that stoichiometrically requires oxygen for degradation [19, 23]. Aroma composition of wines shares a close relationship with must amino acid composition, where volatile compounds such as isoamyl acetate, isobutanol, isobutyric acid and methionol are significantly different among cultivars [24]. Grape composition at harvest can therefore impact the quality of the finished wine.

The environmental influence of water deficit has been positively correlated with the enhancement of quality attributes such as color, aroma and flavor [25, 26]. For example, Deluc et al. [27] investigated seasonal water deficit in Cabernet Sauvignon observing 2-fold increases in the accumulation of the five major anthocyanins, as well as significant increases to the MYB transcription factors that regulate the final steps in anthocyanin biosynthesis. Drought tolerance amongst cultivars also varies between grapevine cultivars and species [28, 29]. Wine produced from low water status vines had significant reductions in vegetal aroma, but were rated highly for fruity aromas associated with red and black fruit [30]. Water-deficit-treated berries also showed significantly induced transcripts involved in fatty acid cleavage or hydroxylation of monoterpenes leading to plant volatile production [31]. Severe water deficit can also increase berry nitrogen status [32] by differentially affecting the transcription of amino acid metabolism, including proline, glutamate and phenylalanine [27].

In the present study, an integrated analysis (transcriptional, translational, and intermediary and end-products of metabolism) is presented to test the uniqueness of three red-skinned and two white-skinned cultivars: Cabernet Sauvignon, Merlot, Pinot Noir, Chardonnay and Semillon, respectively. Here, the same berry samples from the same vineyard and climate, free of disease and insect pressures, were sampled and utilized for each Omic analysis. The cultivars were exposed to a mild, seasonal water-deficit treatment from fruit set until harvest in 2011 to provide a more diverse molecular expression that underlies the unique responses of each cultivar. The major goal of this research was to elucidate the major biochemical and signal transduction pathways that were active at berry maturity. This was accomplished using an Omics approach to identify and quantify the relative abundance of transcripts, proteins and metabolites in the berry skins. Another goal was to evaluate the platform performance of gene expression profiled by NimbleGen Grape Whole-Genome Microarray and Illumina RNAseq technologies. In addition to comparing abundance changes of individual proteins and transcripts, ancillary components of the berry biological system were determined through primary and secondary metabolite analyses using gas chromatography-mass spectroscopy (GC-MS) and liquid chromatography-mass spectroscopy (LC-MS). Interestingly, the cultivars’ proteomic, transcriptomic and metabolomic responses to the drought treatment were divergent, reflecting, at the level of the berry skin, unique grape profiles. In this comprehensive assessment of five grape berry cultivars at harvest, we found that there was concordance amongst the Omics platforms in differentiating each cultivar’s uniqueness.

## Results

Five Omic data sets comprising transcripts, proteins, and metabolites, generated from the same harvested skins, were used to investigate cultivar differences in biochemistry and signal transduction at berry maturity. An emphasis upon known, biologically-important molecules of the mature berry that affect color and amino acid metabolism will be presented here.

### Growth conditions and physiological data

**Fig. 1:**
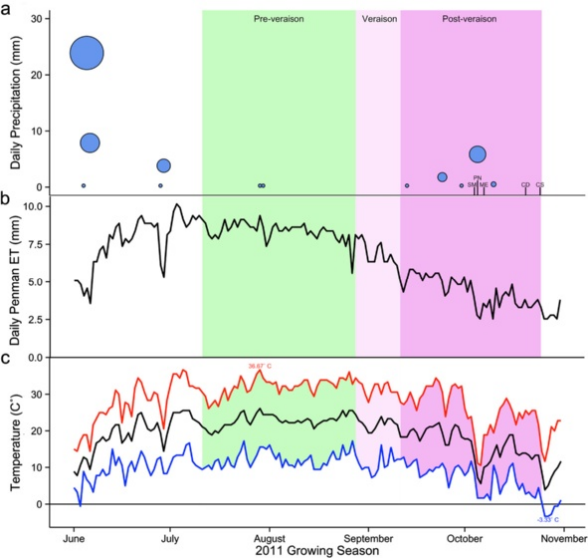
Seasonal precipitation and temperature at the Nevada Agricultural Experiment Station Valley Road Vineyard were collected from the Desert Research Institute’s weather station. The double sigmoidal phases of berry development are highlighted: Pre-veraison in green refers to fruit set and enlargement before color change; Veraison in pink refers to the transition in color of berries; and Post-veraison in purple refers to full color change and heightened sugar and decreased organic acid levels until harvest. Harvest time points in October 2011 are denoted by cultivar abbreviations in their respective order of harvest: Semillon (SM), Pinot Noir (PN), Merlot (ME), Chardonnay (CD), and Cabernet Sauvignon (CS). **a** Daily precipitation (mm) values are illustrated by blue circles, scaled to the amount of precipitation on a given day. **b** The daily total Penman evapotranspiration (mm) values were based on the 82 Kimberly-Penman equation. **c** The daily high (red), low (blue) and mean (black) temperatures and the extreme high (36.7 °C) and low (−3.33 °C) are indicated

Cabernet Sauvignon, Merlot, Pinot Noir, Chardonnay, and Semillon were grown at the University of Nevada, Reno Experimental Vineyard during the 2011 growing season in relatively normal and stress-free conditions. This vineyard is located at high elevation (1372 m) in a very dry climate (see seasonal precipitation (Fig. 1a), estimated daily evapotranspiration (Fig. 1b) and daily mean temperatures (Fig. 1c)). Seasonal precipitation from fruit-set through veraison (July – September) was marginal, totaling 0.501 cm, with daily mean temperatures of 22.5 °C. The majority of rain accumulation occurred late in the season (early October 2011), which also coincided with a period of cooler daily mean temperatures (8.9 °C) and the harvests for Semillon, Pinot Noir, and Merlot. The remaining growing days of the 2011 season maintained warmer temperatures (daily mean 14.3 °C) and an absence of rain. Cabernet Sauvignon fruit were harvested the day prior to the season’s first freezing temperatures (−3.3 °C) in order to avoid potential frost damage to berries.

Grapevines were grown in two adjacent experimental vineyards with independent irrigation controllers. Merlot, Pinot Noir, and Semillon were grown in the experimental south, which had a randomized-block experimental design (see Methods and Additional file 1 for details). Different rows were under different irrigation controls. Drip irrigation was initiated when stem water potentials of the vines reached their target treatment level, stem water potentials (ψ_w_) of −0.6 MPa for control vines and −0.8 MPa for a mild water deficit. Mid-day stem water potentials were monitored weekly for well-watered (WW)- and water-deficit (WD)-treated vines to assess plant water status and to determine the amount of water to be applied to maintain stem water potentials over the season (Table 1; Additional file 2). The water potentials of vines were close to target stem water potentials at the time of harvest.

**Table 1:** Mid-day stem water potentials at harvest time point. Measurements conducted on mature, fully expanded leaves. Values are mean ± SE

Vineyard^a^	Treatment^b^	ψ_w_ (MPa)^b^	n^c^
North	Water deficit	−0.84 (±0.11)	6
	Well watered	−0.61 (±0.03)	8
South	Water deficit	−0.95 (±0.04)	15
	Well watered	−0.68 (±0.04)	14

^a^North = Cabernet Sauvignon and Chardonnay; South = Merlot, Pinot Noir, and Semillon

^b^MPa = megapascal

^c^Inconsistencies between sample size were due to damaged leaves at time of sampling

Berries were monitored weekly from fruit set through to harvest, to assess °Brix and titratable acidity (TA) levels by sampling two average clusters per replicate, cultivar and treatment from two non-adjacent vines. The timing of harvest for each cultivar was determined by berries sampled for a target °Brix to TA ratio of 3.5. The average °Brix and TA (g L^−1^) were 23.3 and 7.1, respectively, with a ratio of 3.3. For each cultivar, WW and WD grape berries were harvested on the same day. Mild water deficit treatment had no significant effect upon berry diameter, °Brix, or TA at harvest (Table 2), with the exception of a 4 % reduction of Pinot Noir berry diameters that was statistically significant at *p* ≤ 0.01. Reported physiological measurements and water deficit levels were similar to data reported by Grimplet et al. [33] in their proteomic analysis of grape berry tissues under water deficit.

**Table 2:** Berry physiological measurements at the harvest time point

Varietal	Treatment^a^	Berry diameter (mm)^b^	°Brix^c^	TA (g l^−1^)^cd^
Cabernet Sauvignon	WW	11.16 (±0.07)	23.11 (±0.20)	8.43 (±0.25)
	WD	11.09 (±0.07)	23.66 (±0.27)	8.42 (±0.29)
Merlot	WW	11.72 (±0.09)	22.99 (±0.23)	5.50 (±0.38)
	WD	11.55 (±0.08)	23.31 (±0.30)	6.06 (±0.43)
Pinot Noir	WW	12.09 (±0.07)	22.85 (±0.46)	5.80 (±0.09)
	WD	11.51 (±0.07)	22.95 (±0.46)	6.12 (±0.24)
Chardonnay	WW	12.11 (±0.06)	23.35 (±0.39)	9.18 (±0.26)
	WD	12.07 (±0.07)	23.42 (±0.25)	8.83 (±0.44)
Semillon	WW	13.47 (±0.09)	23.18 (±0.40)	6.40 (±0.32)
	WD	13.29 (±0.09)	23.82 (±0.33)	6.53 (±0.28)

^a^WW = well watered; WD = water deficit

^b^Measurements conducted on individual berries

^c^Measurements conducted on whole clusters

^d^Expressed in g L ^−1^ tartaric acid

Values are mean ± SE, with n = 3 for berry diameter and n = 6 for °Brix and titratable acidity (TA) measurements. Differences between treatments were determined to be significant (*p*-value < 0.01) by the Student’s *t*-test

### Comparative Omic analyses of grape berry skin

Our comparative Omic analyses focused on the skins, which had been separated from the pulp and seeds of ripe berry clusters at harvest and rapidly frozen in liquid nitrogen. At least two clusters per experimental replicate (six individual vines in total) were harvested in preparation for each sample extraction and analysis (Additional file 1). Berry skins were combined from each experimental replicate and ground and mixed in liquid nitrogen before dividing samples into separate aliquots for chemical extraction. Proteins were extracted from aliquots of three experimental replicates with a modified phenol-based protocol [34], digested with trypsin and Lys-C and analyzed using nanoflow liquid chromatography-mass spectrometry (nanoLC-MS/MS) [35]. Peptide to spectrum matching, protein identification, and normalized spectral abundance factors (NSAF), were computed as described previously [36] (see Methods for details). Approximately 50,000 spectra per sample were assigned to peptides matching a total of 2867 non-redundant *Vitis vinifera* proteins in the UniProtKB database (Table 3; Additional file 3). From the non-redundant proteins, 1211 were shared across all five of the cultivars and had spectra assigned for all experimental replicates (Additional file 4).

**Table 3:** Comparative omic analyses

Data set	n
Proteins (nanoLC-MS/MS)	2867
Transcripts (microarray)	29,549
Transcripts (RNAseq)	27,252
Metabolites measured by GC-MS	67
Metabolites measured by LC-MS	42

Total RNA was extracted with a modified CTAB protocol [37–40] from aliquots of five experimental replicates for NimbleGen (Roche NimbleGen, Madison, Wi) Grape Whole-Genome Microarray analysis, with standard microarray processing and data normalization as in Cramer et al. [41]. Microarray analysis profiled 29,549 genes as predicted in the 12x V1 annotation of the grape genome (Additional file 5). Ground skin sample aliquots from the same three experimental replicates used for the protein analysis were sequenced with an Illumina HiSeq 2000 sequencing system to determine transcript abundance. Transcript data were generated by aligning quality-filtered sequence reads to the grape genome [42], assigning transcript counts to the V1 annotation with the htseq-count tool [43], and then performing a differential expression analysis with the edgeR [44] package (Table 3, Additional file 6). We detected the expression of 27,252 transcripts of the 29,971 transcripts in the V1 annotation.

Metabolites were extracted in parallel from aliquots of all six experimental replicates, three additional replicates from the aforementioned, with a protocol previously described [45]. For metabolite analyses, the peaks of each metabolite were normalized to the total peak area giving a relative metabolic abundance value. The relative metabolic abundance from berry skins of primary and secondary metabolites (Table 3, Additional file 7) were analyzed by GC-MS and LC-MS based methods.

**Fig. 2:**
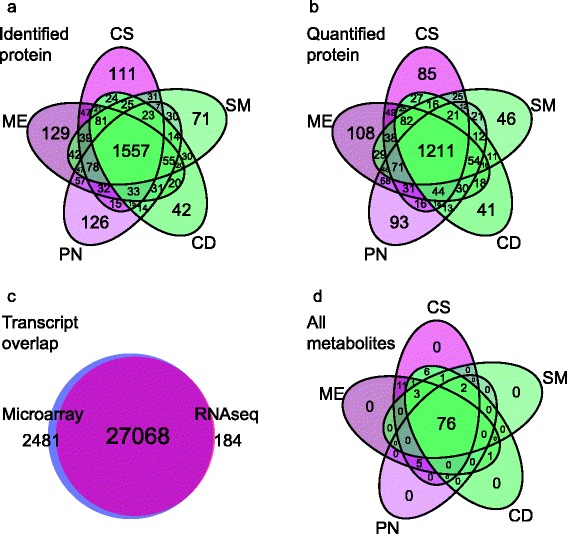
Venn diagrams of the (**a**) identified and (**b**) quantified proteins, the overlap of (**c**) transcripts assessed with either platform, and (**d**) all the metabolites measured in each cultivar, Cabernet Sauvignon (CS), Merlot (ME), Pinot Noir (PN), Chardonnay (CD) and Semillon (SM)

Venn diagrams illustrate the distributions of identified (Fig. 2a) and quantified (Fig. 2b) proteins in the different cultivars. In each case, subsets of proteins were distributed to each cultivar. The majority of transcripts were assessed by both platforms (Fig. 2c). Microarrays measured probe fluorescence for 2481 transcripts that did not receive unique counts by RNAseq. A subset of 1201 transcripts from both platforms could be paired to the quantified proteins. The majority of metabolites were measured in each cultivar (Fig. 2d), with the main metabolite differences attributed to the anthocyanin production in red cultivars.

The most abundant proteins and transcripts from each of the five cultivars were determined. Only proteins detected in all samples (1211) were assessed, but all transcripts measured were considered for this analysis in both platforms. The top ten most abundant proteins (Table 4) surveyed in each cultivar consisted of only 17 proteins, many of which can be classified as pathogenesis-related (PR). Additionally, three of the proteins were in the top ten most abundant of each cultivar: β-1, 3, glucanase [UniProtKB:F6HLL9], major latex protein 22 [UniProtKB:A5BAX1], and a peroxiredoxin-5 [UniProtKB:D7TBK8]. Both transcript platforms were assessed for the degree of concordance in reporting highly expressed transcripts. The top most abundant transcripts by microarray (Table 5) consisted of a common set of 16 uniquely annotated transcripts from the cultivars. Again, several of the top transcripts were PR protein-related including a class IV chitinase, a non-specific lipid-transfer protein and two thaumatins. Five of the transcripts were also ranked in the top ten of each cultivar: invertases/pectin methylesterase inhibitor [UniProtKB:Q9M4H8; EnsemblePlants:VIT_16s0022g00960], chitinase class IV [UniProtKB:Q7XAU6; EnsemblePlants:VIT_05s0094g00340], putative ripening-induced protein 1 [UniProtKB:Q6VEQ6; EnsemblePlants:VIT_05s0049g00760], photosystem II protein D1 [UniProtKB:F6GXB0; EnsemblePlants:VIT_11s0052g01680], and one transcript without a known annotation [UniProtKB:F6H8M1; EnsemblePlants:VIT_05s0049g00520). A BLAST search of the unannotated transcript references a putative proline-rich protein in several species including grape. For RNAseq transcripts (Table 6), a common set of 18 uniquely annotated transcripts made up the top ten from the cultivars. As with the proteins and microarray transcripts, many of the top transcripts were the same PR proteins in the microarrays. Five of the transcripts were also ranked in the top ten of each cultivar: putative ripening-induced protein 1 [UniProtKB:Q6VEQ6; VIT_05s0049g00760], chitinase class IV [UniProtKB:Q7XAU6; EnsemblePlants:VIT_05s0094g00340], abscisic stress ripening protein 2 [UniProtKB:F6GY46; EnsemblePlants:VIT_18s0072g00380], allergenic protein Pt2L4 [UniProtKB:Q9M4H7; EnsemblePlants:VIT_12s0059g00590], and the same unannotated transcript in the microarrays [UniProtKB:Q9M4I2; EnsemblePlants:VIT_05s0049g00520]. Microarray transcripts that did not fully correspond with the RNAseq are annotated as containing probesets that potentially cross hybridize with other closely related genes. For example, all four probes that map to the *cupin* and *Photosystem II protein D1* genes listed in Table 5 have the potential for cross hybridization (see Cramer et al., [41] for a full list of genes with potential hybridization, Additional file 5).

**Table 4:** Top ten most abundant proteins quantified within each cultivar

			Cultivar^b^				
UniProtKB	V1 ID	Annotation^a^	CS	ME	PN	CD	SM
D7TBK8	VIT_11s0016g03630	Peroxiredoxin-5	1	2	10	6	5
F6GY46	VIT_18s0072g00380	Abscisic stress ripening protein 2	2	–	–	5	9
A5BQN6	VIT_03s0038g01930	Peptidyl-prolyl cis-trans isomerase ROC5	3	3	–	–	–
Q9M4H4	VIT_06s0004g02560	Kiwellin Ripening-related protein grip22	4	–	–	–	–
Q9M4H7	VIT_12s0059g00590	Allergenic protein Pt2L4	5	–	–	10	–
F6HUD1	VIT_02s0025g03600	Phospholipid hydroperoxide glutathione peroxidase	6	–	–	–	–
Q7XAU6	VIT_05s0094g00340	Chitinase class IV	7	7	5	–	6
D7SKR5	VIT_06s0004g03550	L-ascorbate peroxidase 1, cytosolic	8	10	–	–	7
F6HLL9	VIT_08s0007g06040	Beta-1, 3-glucanase	9	8	7	8	10
A5BAX1	VIT_01s0011g05110	Major latex protein 22	10	4	8	7	3
F6HUH1	VIT_02s0025g04330	Thaumatin VVTL1	–	1	1	1	2
D7TXF5	VIT_14s0081g00030	Pathogenesis-related protein-4 (Chitinase)	–	5	4	–	4
Q9FS43	VIT_05s0077g01580	Pathogenesis protein 10	–	6	–	–	–
A5C9F1	VIT_02s0025g04300	Thaumatin	–	9	3	4	8
F6HUG9	VIT_02s0025g04310	Thaumatin	–	–	2	2	–
F6HUG6	VIT_02s0025g04280	Osmotin	–	–	6	9	–
F6GXX3	VIT_08s0058g01230	Non-specific lipid-transfer protein	–	–	9	3	1

^a^Annotation by Grimplet et al. [80]

^b^ *CS* Cabernet Sauvignon, *ME* Merlot, *PN* Pinot Noir, *CD* Chardonnay, *SM* Semillon

The number within each cultivar column represents the abundance rank for that cultivar, with the number ‘1’ being the highest

**Table 5:** Top ten most abundant transcripts (microarray) within each cultivar

			Cultivars^b^				
UniProtKB	V1 ID	Annotation^a^	CS	ME	PN	CD	SM
F6H8W9	VIT_12s0034g01970	Cupin	1	1	4	–	–
F6H8M1	VIT_05s0049g00520	Putative uncharacterized protein	2	2	1	1	1
Q9M4H8	VIT_16s0022g00960	Invertase/pectin methylesterase inhibitor	3	3	3	7	6
Q9M4H7	VIT_12s0059g00590	Allergenic protein Pt2L4	4	–	9	9	8
Q7XAU6	VIT_05s0094g00340	Chitinase class IV	5	5	6	8	5
Q6VEQ6^c^	VIT_05s0049g00760	Putative ripening-induced protein 1	6	4	7	2	2
D7SLR0	VIT_15s0021g02700	Beta-expansin (EXPB4)	7	–	–	–	–
F6HFY8^c^	VIT_01s0010g01260	23S ribosomal RNA	8	–	–	–	7
A5B118	VIT_08s0007g03830	fructose-bisphosphate aldolase cytoplasmic isozyme	9	10	–	–	–
F6GXB0^c^	VIT_11s0052g01680	Photosystem II protein D1	10	9	8	5	4
F6HUG9	VIT_02s0025g04310	Thaumatin	–	6	2	3	–
F6HUH1	VIT_02s0025g04330	Thaumatin VVTL1 [Vitis vinifera]	–	7	5	4	10
F6GV13	VIT_06s0004g04650	Metallothionein	–	8	–	6	–
A5C670^c^	VIT_13s0064g01210	Zf A20 and AN1 domain-containing stress-associated protein 2	–	–	10	–	–
F6GXX3	VIT_08s0058g01230	Non-specific lipid-transfer protein	–	–	–	10	3
F6HPX1^c^	VIT_13s0101g00220	Ribosomal RNA 16S	–	–	–	–	9

^a^Annotation by Grimplet et al. [80]

^b^ *CS* Cabernet Sauvignon, *ME* Merlot, *PN* Pinot Noir, *CD* Chardonnay, *SM* Semillon

^c^Not identified in protein data set

**Table 6:** Top ten most abundant transcripts (RNAseq) within each cultivar

			Cultivar^b^				
UniProtKB	V1_ID	Annotation^a^	CS	ME	PN	CD	SM
F6H8M1	VIT_05s0049g00520	Putative uncharacterized protein	1	1	1	1	1
Q6VEQ6^c^	VIT_05s0049g00760	Putative ripening-induced protein 1	2	2	2	2	2
F6HEL0	VIT_19s0090g01370	Putative uncharacterized protein	3	6	–	7	–
F6H8M0^c^	VIT_05s0049g00510	Ethylene response factor ERF1	4	7	7	–	5
Q7XAU6	VIT_05s0094g00340	Chitinase class IV	5	3	3	5	6
F6GY46	VIT_18s0072g00380	Abscisic stress ripening protein 2 (ASR2)	6	4	5	4	3
Q9M4H7	VIT_12s0059g00590	Allergenic protein Pt2L4	7	10	10	9	8
D7T852	VIT_19s0090g01340	Putative uncharacterized protein	8	–	9	–	–
D7T853	VIT_19s0090g01350	Aspartyl protease	9	–	–	–	–
F6GU22	VIT_06s0004g02560	Kiwellin Ripening-related protein grip22	10	–	–	–	–
F6HUH1	VIT_02s0025g04330	Thaumatin VVTL1 [Vitis vinifera]	–	5	4	3	4
Q9M4H8	VIT_16s0022g00960	Invertase/pectin methylesterase inhibitor	–	8	6	–	–
F6GV13	VIT_06s0004g04650	Metallothionein	–	9	–	–	–
F6HUG9	VIT_02s0025g04310	Thaumatin	–	–	8	–	–
F6GXX3	VIT_08s0058g01230	Non-specific lipid-transfer protein	–	–	–	6	10
D7TAI4	VIT_01s0010g02030	Gamma-thionin precursor	–	–	–	8	–
F6HMP0^c^	VIT_08s0056g01600	Putative uncharacterized protein	–	–	–	10	9
D7T2C8	VIT_05s0094g00350	Chitinase class IV	–	–	–	–	7

^a^Annotation by Grimplet et al. [80]

^b^CS = Cabernet Sauvignon; ME = Merlot; PN = Pinot Noir; CD = Chardonnay; SM = Semillon

^c^Not identified in protein data set

A multifactorial (5 x 2; cultivar x treatment) experimental design was used for each platform to determine significant differences (adjusted *p*-value with a false discovery rate < 0.05; herein referred to as “significant” throughout this paper) amongst treatments and cultivars. ANOVA indicated that the cultivar level contributed the largest amount of significant changes in each of the data sets (Table 7). Statistically significant transcript abundance changes were found for both transcript technologies for cultivar, treatment, and cultivar x treatment effects [46]. Neither the treatment effect nor the interaction effect (treatment x cultivar) was statistically significant with the protein or metabolite data, but significant cultivar effects were found with protein and metabolite abundances.

**Table 7:** Statistically significant results from each Omics dataset adjusted for multiple testing using FDR (0.05)

Dataset	Treatment	Cultivar	Cultivar x Treatment
Proteins	0	832	0
Transcripts			
Microarray	195	27,064	1,546
RNAseq	1	15,149	241
Metabolites			
GC-MS	0	63	0
LC-MS	0	40	0

Differential expression analysis of transcripts was similarly performed for both platforms. Standard processing and data normalization of the microarrays was performed. ANOVA indicated transcript abundance of 27,064 transcripts changed significantly with cultivar, the transcript abundance of 195 transcripts changed significantly with treatment, and 1546 transcripts changed with the cultivar x treatment interaction term. RNAseq data were normalized and modeled with the standard edgeR pipeline. Generalized linear models were fit to a multifactorial design formula (5 x 2; cultivar x treatment) for significance testing, and indicated 15,149 transcripts changed significantly with cultivar; the transcript abundance of one transcript changed significantly with treatment; and 241 transcripts changed with the cultivar x treatment interaction term.

There was a common set of 1211 proteins that was quantifiable across each of the cultivars and treatments. This consistent set of proteins was considered for further reliable comparative quantitative analyses. The protein abundance of 832 proteins changed significantly with cultivar (Table 7), but there were no significant changes in protein abundance for either treatment or cultivar x treatment interaction terms. In addition, the relative metabolic abundance of primary and secondary metabolites (Additional file 7) changed significantly with cultivar, but no metabolite abundances were changed significantly for either treatment or cultivar x treatment interaction terms (Table 7).

A comparison of Tables 3 and 7 reveals that the percentage of the transcripts varied substantially with cultivar between the two transcriptomic platforms, 92 % for the microarray platform and 56 % for the RNAseq platform. The percentage of proteins varying with cultivar was approximately 69 % and the percentage of metabolites varying with cultivar was approximately 95 % for both platforms. Thus, all Omic platforms revealed a large variability in molecular abundance amongst all the cultivars.

In summary, data variability was mostly attributed to the cultivar effect. The mild water deficit treatment effect was much less. ANOVA results indicate that mild water deficit did induce a significant change in the abundance of a small percentage (<6 %) of transcripts. Protein and metabolite abundances in this study were significantly affected only by a specific cultivar.

**Fig. 3:**
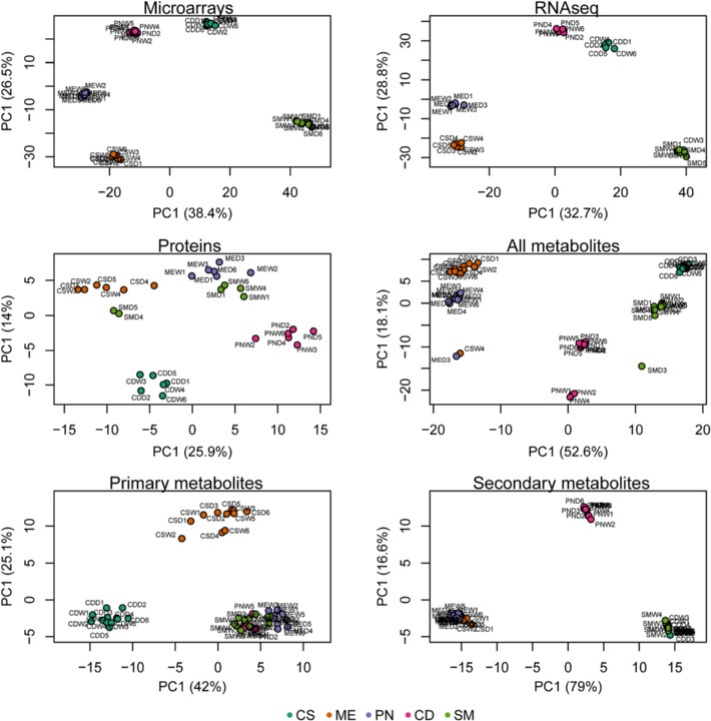
Principal components analysis of each Omic platform. Experimental replicates are labeled and colored consistently in each platform, Cabernet Sauvignon (CS), Merlot (ME), Pinot Noir (PN), Chardonnay (CD) and Semillon (SM). W = Well-Watered; D = water deficit. The following numbers refers to an experimental replicate sample

Experimental samples from each platform were analyzed by principal components analysis (PCA) (Fig. 3), which reduced the dimensionality of the data and allowed a clearer observation of the underlying structure. Each PCA biplot showed the directions where there was the most variance in the data. Cultivars separated from one another similarly on the first principal component in each platform providing substantial concordance amongst the different Omic approaches. Generally, red cultivars separated from white, but Pinot Noir samples separated somewhere in between. Biological variability in samples was evident particularly in protein and metabolite biplots. The secondary metabolites were separated along the first component, separating the red cultivars that synthesize anthocyanins, and anthocyanin moieties separated Cabernet Sauvignon and Merlot from Pinot Noir. Water-deficit and well-watered samples at harvest could not be differentiated clearly in PCAs reflecting the results from the ANOVA.

**Fig. 4:**
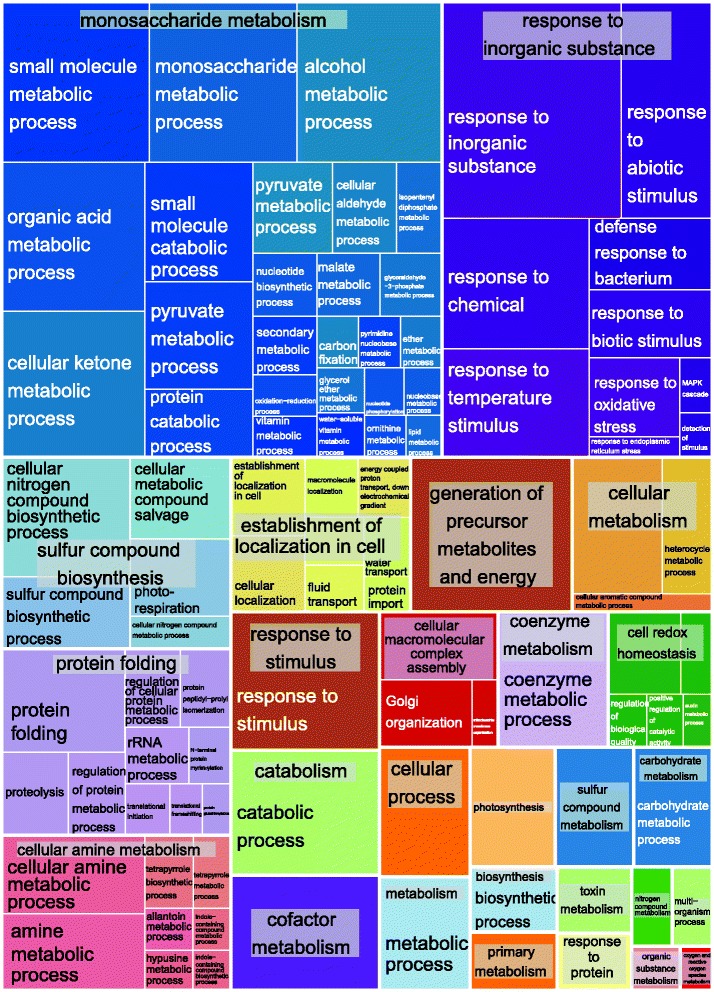
Overrepresented GO biological process terms. The functional analysis of 1211 quantifiable proteins visualizes related terms by color, and rectangles were size adjusted to reflect their enriched *p*-value

A functional analysis (Additional file 8) was performed to identify gene ontology (GO) categories for the quantifiable proteins with the BinGO (3.0.2) plugin for Cytoscape (3.1.1), using a custom annotation derived from UniProt (uniprot.org), EnsemblPlants (plants.ensembl.org), and Gramene (gramene.org) [47, 48]. There were 479 significantly overrepresented GO categories after correcting for FDR (adjusted p-value of 0.05). To aid our analysis, overrepresented GO terms were visualized (Fig. 4) with a treemap using REVIGO and the treemap R package that depicts loosely related GO terms by color [49]. Rectangles in the treemap are size adjusted to reflect their enriched p-value. The functional analysis examined the results both by the level of significance and by the number of constituents of each GO category, in an effort to look beyond generic or overly encompassing functional categories (e.g. metabolic process). Some of the major biological process GO categories for proteins that were overrepresented included organic acid metabolic process, monosaccharide metabolic process, generation of precursor metabolites and energy, alcohol metabolic process, and response to abiotic stimulus.

### Correlations between proteomic and transcriptomic data

**Fig. 5:**
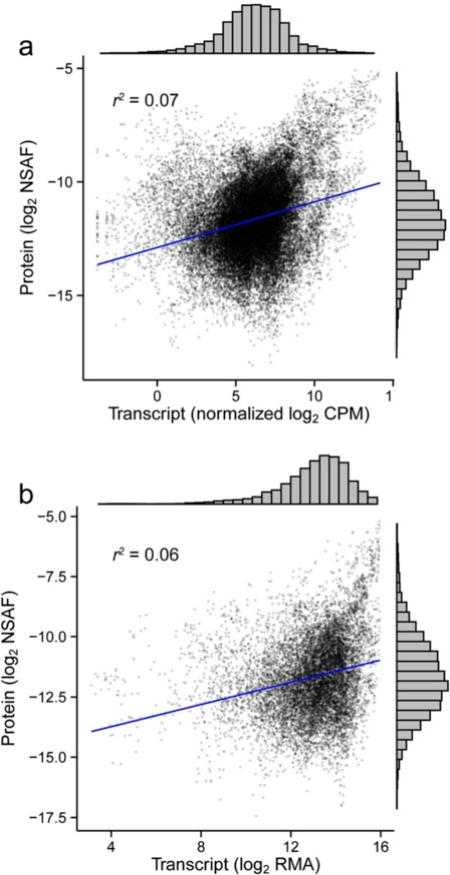
Correlations between log2 transformation of the normalized protein and transcript abundance of five grapevine cultivars. The correlation between 1201 transcript-protein pair abundance levels from either (**a**) RNAseq or (**b**) microarray analyses

To investigate the linear relationship of the relative transcript abundance with relative protein abundance, we fit linear regression models to the transcript-protein pairs and computed Pearson’s correlation. A direct sample-to-sample comparison was performed for the RNAseq using the same experimental replicates as were used in the proteomics. The microarray analysis contained two additional experimental replicates for each treatment and cultivar, preventing a direct one-to-one comparison between replicates; thus, mean expression (transcript) and abundance (protein) values were computed for each treatment and cultivar prior to regression analysis. When the transcriptomic and proteomic abundance values were compared for all transcript-protein pairs by a single linear regression (see Additional file 9 for individual), the goodness of fit or coefficient of determination was low (*r*^*2*^ = 0.07 for RNAseq; *r*^*2*^ = 0.06 for microarray); a small positive correlation between the pairs was observed (Pearson correlation coefficient = 0.27 and 0.24 for protein abundance with RNAseq and microarray abundance, respectively) (Fig. 5). Regression with only the top 10 % most abundant proteins increased the correlation coefficient to 0.4 with the RNAseq data, whereas use of the lower 90 % of the abundant proteins reduced the correlation coefficient to 0.14 (data not shown). There was essentially no correlation (0.00) with the RNAseq data and the least abundant proteins of 50 % or less. With the top ten most abundant proteins in the skins (actually 14 proteins for all five cultivars; see Table 4), the number of proteins with significant positive correlations increased to 57 % (8 of 14) and 71 % (10 of 14) for the microarray and RNAseq data, respectively (see Additional file 9 for individual details). Thus, there was an increase in correlation coefficient with increasing protein abundance, but for the majority of the proteins the correlation was very low.

**Fig. 6:**
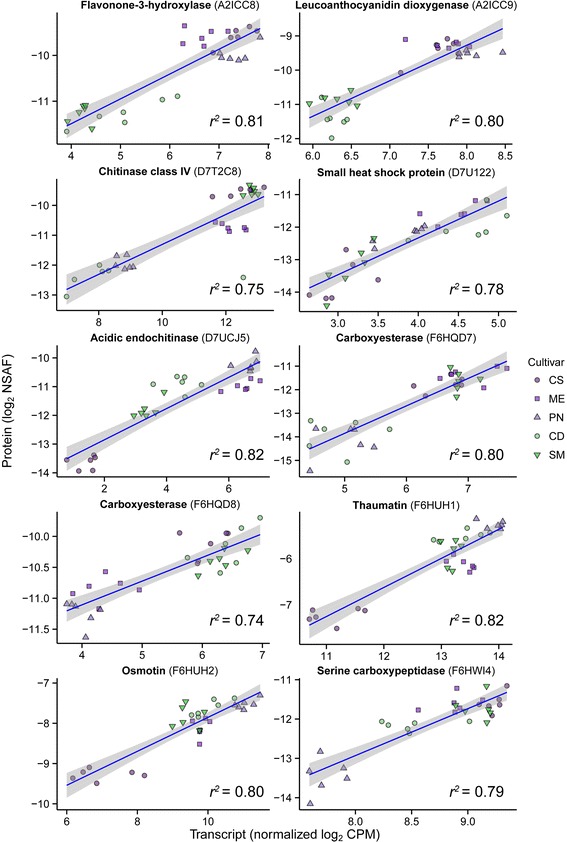
Individual correlations between ten of the highest correlated protein-transcript pairs. Linear regressions and Pearson’s correlation of RNAseq and protein data sets were direct sample-to-sample comparisons. The UniProtKB accession number of each protein is given in parentheses

Some proteins with strong positive correlations included pathogenesis-related proteins, carboxyesterases and proteins related to phenylpropanoid and flavonoid production (Fig. 6); all of these proteins had a minimum Pearson correlation coefficient of 0.86 (r^2^ = 0.74). Generally, protein-transcript pairs grouped together by cultivar and occasionally by skin color. Stronger negative correlations were observed with the microarray data (−0.93) than with RNAseq data (−0.68). Protein-transcript pairs with strong negative correlations included a translation initiation factor eIF3 subunit (*r*^*2*^ = 0.41, Pearson correlation coefficient = −0.67; [UniProtKB:D7TMG2, EnsemblePlants:VIT_13s0019g03470]) and a chlorophyll A-B binding protein (*r*^*2*^ = 0.46, Pearson correlation coefficient = −0.68; [UniProtKB:A5BPB2, EnsemblePlants:VIT_12s0028g00320]), a constituent of the light-harvesting complex. Other negatively correlated protein-transcript pairs included several heat shock proteins and a putative serine/threonine kinase.

Overall, for the abundance of the 1201 proteins identified, 24 and 33 % had a significant correlation with their transcript abundance for microarray and RNAseq data, respectively. These data sets of significantly correlated protein-transcript pairs were analyzed for overrepresentation of functional categories using the GO categories for biological processes (data not shown). There were many functional categories overrepresented, however, these same categories were overrepresented also in the full set of 1201 proteins. Additionally there were no substantial changes in the percentages of each functional group in their respective data sets. Thus, the correlation of the protein-transcript pair appears to be independent of their functional category.

### Transcriptomic platform concordance

**Fig. 7:**
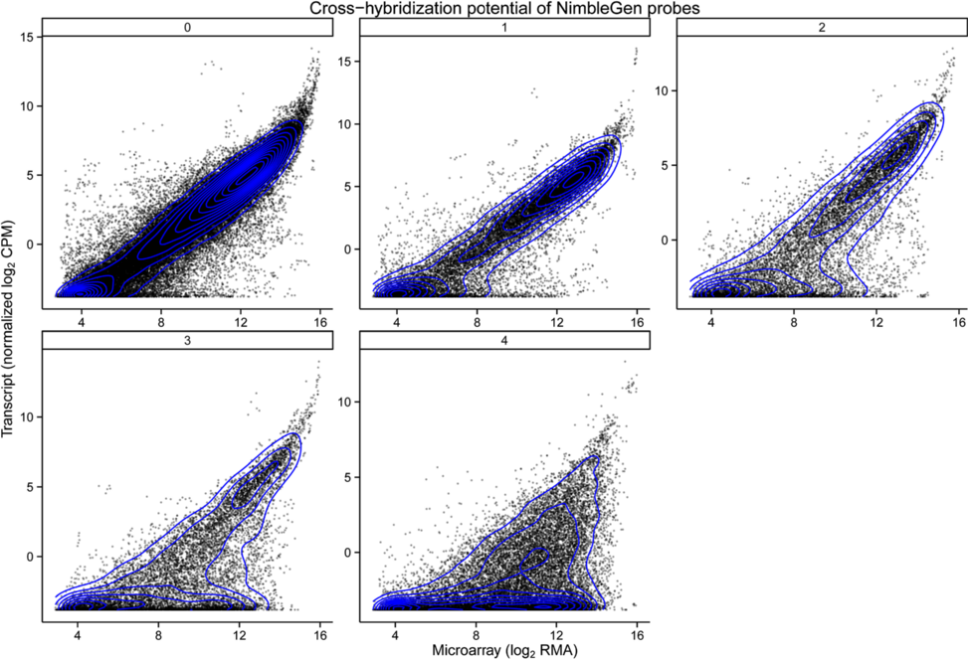
Pairwise platform comparisons of measured transcripts. Each subfigure (0, 1, 2, 3 or 4) represents the RNA-seq vs Microarray data of the transcripts that are subset by the number of probes (0, 1, 2, 3 or 4) with cross-hybridization potential on NimbleGen microarrays. Transcript expression values are the average treatment and cultivar expression level due to unequal experimental replicates between platforms, n = 3 for RNAseq and n = 5 for microarrays

The protein-transcript pair comparison indicated that a larger number of proteins were more significantly correlated with the RNAseq data than the microarray data. Therefore, we measured how similar the two different platforms, open (RNAseq) and closed (microarray), measured gene expression levels by Pearson correlation and linear regression, on a gene-by-gene basis. In Cramer et al. [41], we cautioned readers about the likelihood of cross-hybridization potential of approximately 13,000 genes on the NimbleGen Grape Whole-Genome microarray. Many of these transcripts belong to *Vitis* gene families with high sequence similarity that creates an opportunity for at least one probe from a set of four probes to cross-hybridize with probes from another gene on the array. A global comparison of measureable transcripts shared between the methods presented an opportunity to investigate their concordance. In Fig. 7, a pairwise comparison of each platform’s transcript expression was separated into subsets by the number of probes with the potential for cross-hybridization (0, 1, 2, 3 or 4 probes). Platforms were positively correlated as a whole (Pearson’s correlation coefficient 0.80), but the correlation decreased when examining subsets of transcripts based on the number of probes that cross-hybridize (Table 8). In particular, lowly expressed transcripts in the RNAseq dataset had a variable range (high to low) of expression values measured by microarray.

**Table 8:** Probesets (1 to 4) with potential for cross-hybridization

Probe count^a^	Coefficients^b^	Number of transcripts	Paired-to-protein
0	0.93	15,945	830
1	0.91	3280	177
2	0.83	2061	101
3	0.69	2036	63
4	0.51	3746	30

^a^Flagged transcripts from Cramer et al. [41]

^b^Correlation between RNAseq & microarray

Pearson’s correlation of transcripts annotated for cross-hybridization potential. Affected transcript counts for all transcripts and the subset paired with protein data

### Pathway Omic analyses

To gain a better understanding of the biochemical processes in the mature berry skin and to emphasize how differentiated the cultivars were at harvest, we mapped our Omic data sets to two important biochemical pathways for further analysis. We used the quantifiable protein data as a framework for each map and their matching transcripts. Additionally, metabolite intermediates and final products were also mapped, including amino acids, flavan-3-ols, and anthocyanins. Each pathway summarizes abundance differences depicted as side-by-side heat maps that display the ratio of the individual cultivars average to the overall cultivars average abundance for each data point. The Omic data were overlaid onto customized metabolic pathway maps based upon annotated maps located at KEGG [50], PlantCyc [51], and VitisCyc [52]. Mapped enzymes without heat maps did not contain protein data.

### Differences in phenylpropanoid through anthocyanin biosynthesis

**Fig. 8:**
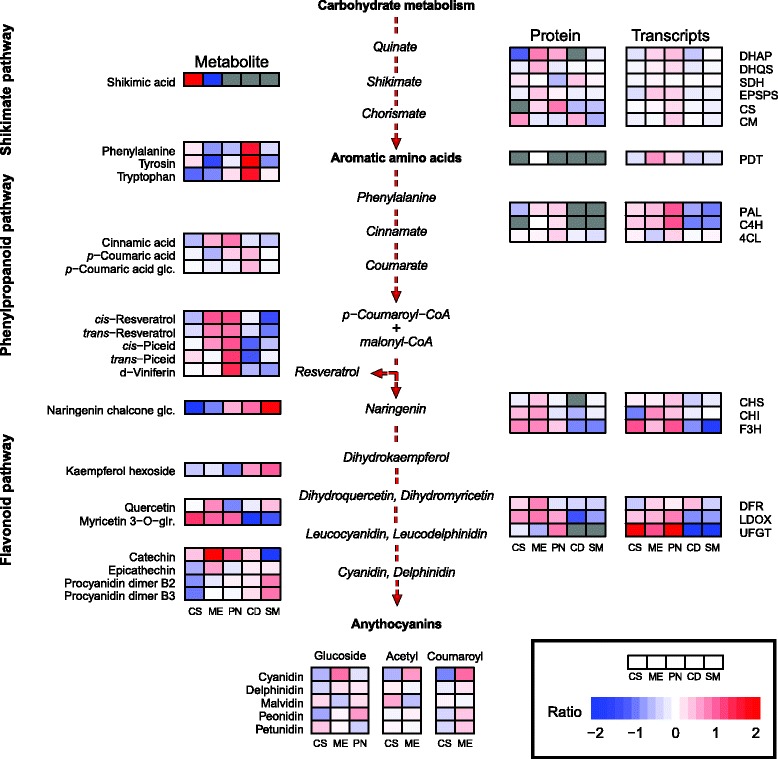
The expression of three Omic data sets in a simplified phenylpropanoid pathway from carbohydrates to anthocyanins. The relative abundances of transcripts, proteins and metabolites are displayed as colored boxes. Relative abundance ratios are of the cultivar average relative to the average of all cultivars. Only transcripts (RNAseq) paired to proteins are shown. The five anthocyanidins measured are organized into rows (anthocyanidin) and columns (glycosylated, acetylated and coumaroylated moieties). Results were derived from experimental replicates (n = 3 for proteins, n = 3 for transcripts, and n = 6 for metabolites). Proteins and metabolites absent for a specific cultivar are colored grey. Cultivar order is from left to right: Cabernet Sauvignon (CS), Merlot (ME), Pinot Noir (PN), Chardonnay (CD), and Semillon (SM). Enzymes and transcripts are given as EC numbers: 3-deoxy-7-phosphoheptulonate synthase (DHAP, 2.5.1.54), 3-dehydroquinate synthase (DHQS, 4.2.3.4), shikimate dehydrogenase (SDH, 1.1.1.25), 3-phosphoshikimate 1-carboxyvinyltransferase (EPSP, 2.5.1.19), chorismate synthase (CS, 4.2.3.5), chorismate mutase (CM, 5.4.99.5), prephenate dehydratase (PDT, 4.2.1.91), phenylalanine ammonia-lyase (PAL, 4.3.1.2.4), trans-cinnamate 4-monooxyygenase (C4H, 1.14.13.11), 4-coumarate-CoA ligase (4CL, 6.2.1.12), chalcone synthase (CHS, 2.3.1.74), chalcone isomerase (CHI, 5.5.1.6), flavanone 3-hydroxylase (F3H, 1.14.11.9), dihydroflavonol 4-reductase (DFR1.1.1.219), leucoanthocyanidin dioxygenase (LDOX, 1.14.11.19), and UDP glucose:flavonoid 3-O-glucosyltransferase (UFGT, 2.4.1.115)

A large number of proteins, transcripts and metabolites could be mapped in the phenylpropanoid pathway (Fig. 8). There was a loose correspondence of proteins and transcripts from red-skinned grapes with their metabolites. We primarily observed higher protein abundance in the red cultivars for enzymes involved in phenylalanine through anthocyanin biosynthesis, such as flavanone 3-hydroxylase and leucoanthocyandin dioxygenase. Missing spectra within the experimental replicates of the white cultivars was evidence of their lesser abundance. Relative to the red cultivars, Chardonnay and Semillon proteins involved in phenylpropanoid and flavonoid were less abundant, although, a chorismate mutase (CM) in Chardonnay was an exception to that observation. Chorismate is an important precursor that interfaces the biosynthesis of phenylalanine and tyrosine, tryptophan, folate, and phylloquinone [53]. Four phenylalanine ammonia-lyases (PAL; [UniProtKB:A5BPT8, F6HNF5, F6HR33, F6HS12]) were identified only within the red-skinned cultivars. Phenylalanine ammonia-lyases (4.3.1.24) are a multigene enzyme family encoding the first committed step in phenylpropanoid biosynthesis [54]. Chalcone synthase (CHS; 2.3.1.74) and stilbene synthase (STS; 2.3.1.95) enzymes both catalyze reactions that condense the substrates 3-coumaroyl-CoA and three malonyl-CoA units in production of flavonoids and stilbenoids, respectively. Three grapevine chalcone synthases [UniProtKB:A2ICC5, F6H419, Q8W3P6] were identified within the proteomic data set [55]. UDP glucose:flavonoid 3-O-glucosyltransferase (UFGT; 2.4.1.115) proteins were observed only in the red cultivars. They catalyze the O-glycosylation of anthocyanidins or anthocyanins that enhance the stability and hydophilicity of anthocyanins *in planta* [56–58]. Of the proteins quantified in each cultivar, all but 3-dehydroquinate synthase (DHQS; 4.2.35) were significantly present at the cultivar level.

In contrast, the transcripts of differentially expressed genes (DEGs) between cultivars in the phenylpropanoid pathway were generally few and occurring after naringenin chalcone in the pathway. More evident were differences between red and white cultivar DEGs of enzymes that centered on flavonoid and anthocyanin biosynthesis such as chalcone synthase, flavanone 3-dioxygenase (F3H; 1.14.11.9) and UDP glucose:flavonoid 3-O-glucosyltransferase (UFGT, 2.4.1.115). These three enzymes had the most abundant transcripts mapped, and are similar to the gene expression for all cultivars but Merlot (not measured) in Boss et al. [57]. No members of the multi-gene stilbene synthase family were detected in the proteomic data set, but one stilbene synthase (*VviSTS3*) encoding transcript (in microarrays) was significantly changed under the interaction term [UniProtKB:F6HIR8; EnsemblePlants:VIT_10s0042g00880], with Cabernet Sauvignon experiencing a −1.6 fold decrease in expression as a result of water deficit [59, 60]. However, *VviSTS3* was lowly expressed in microarrays (1-probe with cross-hybridization potential) relative to other transcripts and contained few counts in RNAseq. Only the UDP glucose:flavonoid 3-O-glucosyltransferase transcript [UniProtKB:D7T7R5; VIT_16s0039g02230] was significant at the treatment level in the microarrays, but each transcript, with the exception of the shikimate dehydrogenase, was significant at the cultivar level.

Both primary and secondary metabolites were measured for each cultivar. Shikimate was among the most abundant metabolites in Cabernet Sauvignon. Aromatic amino acid biosynthesis stems from this intermediate product within the shikimate pathway [53]. Phenylalanine, tryptophan and tyrosine amino acids were recovered in each cultivar. Stilbenoids were also recovered to include *cis-* and *trans-*resveratrol, their glucosides and the polymerized δ-viniferin. Catechin and epigallocatechin, two flavan-3-ol monomers, and procyanidin dimers B2 and B3, consist of two molecules of (+)-catechin or (−)-epicatechin respectively. Flavan-3-ols co-localize with anthocyanins in the hypodermal cells of the berry skin, comprising a diverse and highly abundant class of soluble phenolic compounds [61]. The astringent mouth feel sensations experienced in red wines are derived from these phenolic compounds, with increasing concentrations associated with quality wines [13].

Given that most observable protein and transcript ratio changes were centered at the end of anthocyanin biosynthesis, we present the relative abundance of these metabolites for the three moieties of anthocyanins that were determined (Additional file 10). The importance of color to the sensory experience of red wines is derived from the red, purple and blue anthocyanin pigments produced in the berry skin. Observable differences of anthocyanidin content and their glycosylated, acetylated and coumaroylated moieties amongst the red cultivars were strongly cultivar dependent. All metabolites were significantly different at the cultivar level except malvidin 3-O-(6-p-coumaroyl)glucoside and petunidin 3-O-(6-acetyl)glucoside. Malvidin 3-glucoside had the largest relative abundance of any anthocyanin, and the acetylated and coumaroylated forms of malvidin were also in high abundance in Cabernet Sauvignon and Merlot relative to the other four anthocyanins. Mild water deficit did not have any significant effects on anthocyanin abundance in any cultivar. Thus, all of the variation in metabolite composition could be attributed to the cultivar and not to water deficit.

### Differences in amino acid metabolism

**Fig. 9:**
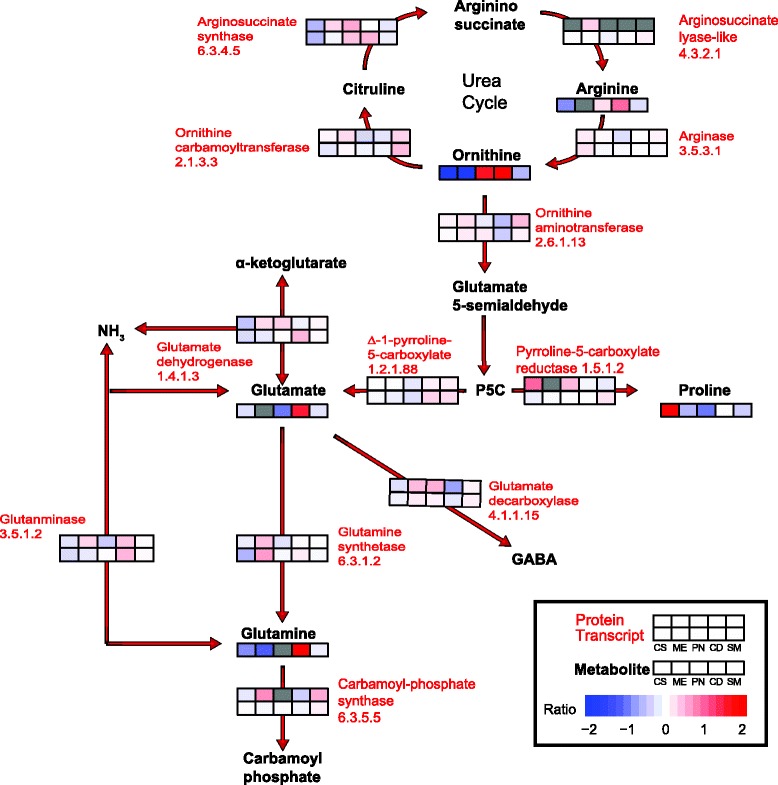
Comparative analysis of three Omic data sets related to amino acid metabolism. The relative abundances of transcripts, proteins and metabolites are displayed as colored boxes. Abundance ratios are of the cultivar average relative to the average of all cultivars. Only transcripts (RNAseq) paired to proteins are shown. Cultivar order is from left to right Cabernet Sauvignon (CS), Merlot (ME), Pinot Noir (PN), Chardonnay (CD), and Semillon (SM). Results were derived from experimental replicates (n = 3 for proteins, n = 3 for transcripts, and n = 6 for metabolites). Proteins and metabolites absent for a specific cultivar are colored grey. Enzymes and transcripts are given as EC numbers: ornithine carbamoyltransferase (2.1.3.3), argininosuccinate synthase (6.3.4.5), argininosuccinate lyase (4.3.2.1), arginase (3.5.3.1), ornithine aminotransferase (2.6.1.13), pyrroline-5-carboxylate reductase (1.5.1.2), L-glutamate gamma-semialdehyde dehydrogenase (1.2.1.88), glutamine synthetase (6.3.1.2), glutamate dehydrogenase (1.4.1.3), glutaminase (3.5.1.2), carbamoyl-phosphate synthase (glutamine-hydrolyzing) (6.3.5.5), and glutamate decarboxylase (4.1.1.15). Abbreviated products and intermediaries: γ-aminobutyic acid (GABA) and 1-pyrroline-5-carboxylate (P5C)

The mature grape berry, via pressed must, provides a source of nitrogenous substances in the form of free amino acids and cleaved peptides, proteins and nucleic acid derivatives, and in mineral ammonium salts that collectively make up the fermentable nitrogen metabolized by yeast during alcoholic fermentation [62]. There was greater correspondence of the mapping of transcripts and proteins with the relative abundance of amino acids as compared with the phenylpropanoid pathway (Fig. 9). Three glutamine synthetases [UniProtKB:A5AP38, D7T6P4, and P51119] were identified in each cultivar; glutamine synthetase is an important enzyme for the condensation of glutamate and ammonia into glutamine. Glutamine synthetases (6.3.1.2) aid in berry nitrogen incorporation [33] and were the most abundant of the enzymes related to amino acid metabolism in each of the five cultivars, with hundreds of peptides identified in each experimental replicate. Of the mapped proteins quantified in each cultivar, all but ornithine aminotransferase (2.6.1.13) and ornithine carbamoyltransferase (2.1.3.3) were significantly different. Transcript abundance differences between cultivars were muted, with the exception of an argininosuccinate lyase (4.3.2.1). Only the arginase (3.5.3.1) transcript [UniProtKB:D7U7W7; EnsemblePlants:VIT_15s0048g00420] in the microarray was significant for the cultivar x treatment term, but all transcripts were significant for cultivar. Chardonnay contained the highest amount of each mapped amino acid (arginine, glutamate, glutamine, ornithine, and proline), except for proline, which was highest in Cabernet Sauvignon (Fig. 9 and Additional file 7). Proline was also the most abundant amino acid quantified by GC-MS. The relative abundance of proline more closely corresponded with the protein abundance of pyrroline-5-carboxylate reductase, but did not correspond with the transcript abundance. The relative abundance of arginine was not significantly different in any measured cultivar, but the abundances of glutamine, glutamate, ornithine and proline were significantly different between cultivars. The higher relative abundance of ornithine, glutamate and glutamine in Chardonnay corresponded to a higher relative transcript abundance of arginosuccinate synthase and glutamine dehydrogenase. The amino acids, glutamate and glutamine, are important sources of available nitrogen for yeast fermentation [1].

## Discussion

The experimental design in this study allowed for a very powerful set of comparative analyses. First, all berry tissues were sampled from the same vineyard site, with vines exposed to the same environment, with nearly identical climate, water and soil (terroir). Second, studying five cultivars further allowed for phenotypic variation of berry metabolism at harvest to be assessed [63]. Third, the Omic analyses benefited from using aliquots of the same tissue, allowing us to better correlate changes between the proteome and transcriptome and observe variations in intermediates and end-products of metabolism. Finally, the power of two transcriptomic methods, closed and open platforms, provided an opportunity to examine potential cross-hybridization events of repeat elements, such as closely related gene family members.

### Omic analyses

While previous proteomic analyses have investigated the proteome of grape berry skin [33, 64–66], our approach estimated protein abundance changes by label-free quantification using spectral counting. A recognized challenge in quantitative proteomics stems from missing data values across replicates for a variety of reasons [67]. Despite the high dynamic range for identifying large numbers of proteins, current label-free proteomic methods are disadvantageous for the detection and quantification of low abundant proteins [68, 69]. Nevertheless, the proteomic results from this study did provide further insight into hundreds of proteins residing within a mature berry skin at harvest in three red and two white cultivars.

Transcript profiling of grapevine was used to assess specific interactions related to cultivar or treatment affects. Both whole and incomplete genome microarrays have been previously utilized in research [27, 31, 41, 70–72] to investigate berry development and the effects of water and salinity stress in both vegetative and berry tissues. For example, a recent investigation of berry pulp and skin revealed a dynamic and active ripening process occurring in the late stages of berry development, with ethylene signaling appearing to play a bigger role in non-climacteric fruit ripening than previously thought [41]. Transcriptionally, the mature berry was very active, and this was evident with the number of transcripts significantly changed for each factor and interaction term. In a previous study, water deficit had significant effects in the fruit of Chardonnay and Cabernet Sauvignon, revealing distinct effects on transcript and metabolite abundance in the pathways for ABA, isoprenoid and stilbene biosynthesis [27, 72]. In our study, only transcription was sensitive enough to detect a significant treatment effect, probably due to the mild treatment level. In addition, the use of five experimental replicates in the microarrays and the detection accuracy of the RNAseq may have increased the ability to detect significantly changing transcripts. Nevertheless, the transcript data presented here offers a rich data set of cultivar differences at harvest that can be used in the future.

Other grape researchers have used high-throughput expression profiling technologies to globally characterize gene expression [59, 73–75]. Dal Santo et al. [76] examined the phenotypic plasticity of Corvina berries from the three most important wine regions around Verona, Italy at various stages of development that revealed a number of non-plastic genes that display stage-specific expression increases or decreases irrespective of vineyard, such as PR and photosynthesis-related transcripts. The observation of non-plastic transcriptome programming partly explains the strong presence of the PR proteins detected in our analysis that accumulate as a disease-prevention strategy.

Comparative Omic analysis has also been used to thoroughly investigate specific metabolic pathways, similar to the metabolic profiling done in this study. Profiling of Sauvignon Blanc with whole genome microarrays [77] putatively identified forty-two carotenoid biosynthesis genes that updated our understanding of one pathway responsible for flavor and aroma production in grapes.

More recently, the measurement of individual gene expression using RNAseq technologies have been used to further our understanding of the transcriptome and are greatly benefited by the higher dynamic range for detection of expression. With unprecedented sensitivity, Zenoni et al. [78] were the first group to utilize RNAseq to profile grape gene expression through berry development; with this approach they were able to identify differential splicing activity and single nucleotide polymorphisms. The observation of unique reads that did not directly map to the reference genome was particularly interesting, further highlighting the power of RNAseq. For example, *de novo* assembly of the Corvina transcriptome [79] revealed 180 new or unique genes (the authors referred to them as private genes) not annotated in the PN40024 reference genome [42]. RNAseq has also been used to describe the expression of specific transcription factors over-expressed at single developmental stages, such as those belonging to the ERF, WRKY and UPBEAT transcription factor families [73]. Knowledge of the timing of transcription factor activity can be used for generating new hypotheses for investigating the regulation of berry development. Collectively, these studies have assisted in furthering our understanding of grapevines and improving the functional annotation of the genome [80]. These transcription studies are very powerful, often for the information not mentioned explicitly in the text but contained in their corresponding data sets.

The availability of the grape genome coupled with microarray and next generation sequencing technology allows global gene expression profiling. Platform concordance was informative of how well each of the technologies performed at measuring transcript abundance. Similar workflows were used beginning with identical tissue and methodology for total RNA extraction and quality assurance checks [81]. Samples also went through similar cDNA syntheses prior to hybridization or library preparation. Closed platforms like microarrays are not readily adapted to improvements made to genomes as are gff3 annotation files and suffer from potential cross-hybridization events. Figure 7 illustrated the decrease of concordance between the platforms for annotated genes with the potential for one or more probe cross-hybridizations. Many of the lowly expressed transcripts in the RNAseq were not accurately modeled in the arrays with a wide range of expression values. The dynamic range of detection was not as high in the microarrays, evident by the right-tail in the pairwise plots. But, the expression profiles of the arrays did follow the relative abundance levels of transcripts seen with RNAseq.

Read numbers per gene are a function of the expression level of the gene, the number of reads generated by the technology and the length of the transcript for those reads to align with. Inefficiencies in measuring gene expression can be related to the degree of read mapping due to poor or incomplete annotations, and RNA that is lost during extraction, or during cDNA conversion and ligation to adaptors. Ultimately, measuring mRNA levels is only a proxy for protein abundance, which is even more complicated when considering the importance of post-translational modifications that affect protein or enzyme activity. While the two transcriptomic platforms were highly correlated with each other, neither platform was an accurate predictor of protein abundance in general. The finding that the abundance of most transcripts is not correlated with the abundance of proteins from the same gene is consistent with a classic study in yeast [82] and many other findings in plants [83–87]. Like the yeast study of approximately 100 proteins [82], our study of approximately 1200 proteins indicated that there was an increased correlation coefficient if one enriched the data set with more abundant proteins. Nevertheless, even in the top 10 % of the most abundant proteins, the abundance of the majority of the proteins did not correlate with its transcript abundance, with less than 50 % of the proteins being significantly correlated with their transcript partner.

### Model assessment and correlation

Why is the relationship of protein abundance to transcript abundance low? Regulation of gene expression can be controlled at many different stages, which may explain partly the poorly observed correlation [45, 79]. For example, transcriptional and post-transcriptional regulation related to the processing of RNA (e.g. alternative or differential splicing) and the stability of the RNA itself can determine the level of expression, where tissue specificity or stress response determines a specific isoform [88]. The general translation of mRNA into protein can also be affected by translational regulation from different regulatory elements (e.g. depletion of ternary complex or hormone signaling) [89, 90]. Protein stability (often measured as a half-life) might also be influenced by the specific isoform or by the conditions that lead to its formation. These examples do not even include the potential for post-translational modifications of the protein [91], which only increases the complexity and reduces the probability for a high correlation of transcript abundance with protein abundance. Yet, subsets of different transcript-protein pairs were strongly correlated, particularly some pathogenesis-related proteins in the skin. At least in the mature berry, the regulation of these genes appears to be tightly controlled at levels upstream of translation.

Transcript-protein pair relationships that lack any correlation can also reveal insights into the biology shared across cultivars. For example, three of the top most abundant proteins quantified [UniProtKB:D7SKR5, EnsemblePlants:VIT_06s0004g03550; UniProtKB:F6HUD1, EnsemblePlants:VIT_02s0025g03600; UniProtKB:D7TBK8, EnsemblePlants:VIT_11s0016g03630] assist in scavenging H_2_O_2_ and are involved in ascorbate-glutathione metabolism; they can offer protective qualities to a maturing berry, irrespective of cultivar, and benefit vine fitness [92, 93]. Both the protein and transcript abundances of ascorbate peroxidase and a glutathione peroxidase were high in each of the cultivars. These data support the hypothesis that high protein abundance levels at this berry developmental stage are important for sustained H_2_O_2_ scavenging and antioxidant activities.

### Effects on berry skin phenolics at harvest

Phenylpropanoids, derived from phenylalanine, are a diverse class of secondary metabolites and are important factors that influence antioxidant activities in grapes and wines. The biosynthesis of small molecular weight phenolics, such as caffeic acid and caftaric acid, peak around the onset of ripening (veraison) and then decrease in the weeks thereafter [75]. As in Castellarin et al. [94], we wanted to link observable changes in our transcriptional and translational data sets with changes in metabolism following a seasonal water deficit. In the present study, numerous protein-transcript pairs and metabolites involved in phenylpropanoid biosynthesis were mapped (Fig. 8), showing the phenotypic diversity of various organoleptic properties (e.g. color and astringency) and berry biochemistry. Enzymes related to anthocyanin biosynthesis were highly abundant relative to other enzymes mapped. Similarly, Deytieux et al. [64] observed high relative abundance of chalcone synthase, flavanone 3-hydroxylase and UDP glucose:flavonoid 3-O-glucosyltransferase enzymes that initiate the gradual accumulation of these phenolic compounds.

Many of the phenylpropanoids were among the most abundant metabolites measured, with the genotype determining the abundance distributions. Metabolic profiling of anthocyanins in the three red cultivars revealed variation in the relative metabolic content of each selected metabolite (Additional file 10). Our results for high levels of malvidin were consistent with those reported previously for Cabernet Sauvignon [45], Malbec [95] and Yan73 (Muscat Hamburg x Alicante Bouschet) [96]. The strong effect of cultivar was evident in protein and metabolite differences observed between the cultivars.

Stilbene abundance also varied between cultivars when compared at harvest. In Cabernet Sauvignon and Shiraz fruit, levels of *trans*-resveratrol accumulated at similar levels from veraison to maturity, whereas its glucoside, *trans*-piceid only increased in Shiraz [45]. Similarly, our cultivars displayed divergent stilbene levels at harvest, with the highest levels observed in Pinot Noir. This is consistent with two comprehensive studies of cultivar comparisons of stilbene concentrations [97, 98], in which Pinot Noir was the cultivar that had the highest stilbene concentrations. Under more severe water deficit, *trans*-piceid metabolite abundance increases 5-fold along with increasing steady state transcript abundance in Cabernet Sauvignon, but not in Chardonnay [72]. These observations are further supported by a 3-year survey of 78 Italian red, white and pink grape cultivars, where large variability in stilbene abundance was consistent with gene expression analysis in the healthy, developing grape berries [97]. The abundance of different stilbenes, like other phenylpropanoids, can distinguish one cultivar from another.

### Importance of assimilable nitrogen in berry skins

Assimilable nitrogen within grape must (fermenting juice) can be a limiting factor to yeast growth during fermentation [22]. The total nitrogen content is distributed primarily in skins and seeds of ripe berries, with the amino acid content ranging from 30 to 40 % depending upon cultivar [1]. Proline, arginine, glutamine, alanine, and glutamate are the major amino acids in fresh grape juice, but the specific composition and concentration of amino acids varies by cultivar, vineyard location and winemaking practices [19, 22, 65]. By sampling and processing berry tissues from the same experimental vineyards, we hoped to reduce some of the variability introduced in our previous studies where the metabolisms of Cabernet Sauvignon and Chardonnay were compared from grapes grown in different geographic locations, root stock and trellis systems [27]. Transcripts related to glutamine and glutamate metabolism were significantly different between cultivars. The metabolite abundance for these two amino acids in this study was low, and reflected different cultivar distributions (Fig. 9). Levels of glutamine and glutamate abundance decrease overtime from veraison to maturity in studies located in Israel and Australia [45, 99]. Proline is one of the major amino acid constituents in both juice and wine, and is formed from 1-pyrroline-5-carboxylate [19, 20, 100]. In two studies, Chardonnay, Cabernet Sauvignon and Shiraz berry skins showed large increases in proline relatively late in the ripening process (post-veraison) peaking at maturity [45, 99]. High proline abundance was observed in each of the cultivars in our study. Ornithine, derived from the urea cycle, can function as a substrate for further amino acid biosynthesis when converted to glutamate 5-semialdehyde (2.6.1.13) by ornithine aminotransferase, which links proline and arginine metabolism [65]. Non-protein amino acids like ornithine and γ-aminobutyric acid (GABA) also contribute to total available nitrogen content within grape must [20]. Bach et al. [101] observed varying GABA concentrations amongst 21 cultivars that changed with region, cultivar and year of harvest, observing the highest GABA levels in Chardonnay. We did not directly measure GABA in this study, but we can hypothesize that GABA levels like other nitrogen contributing compounds measured in this study varied with the cultivar.

### Minor effects of water deficit

Water deficit treatment did not significantly alter the abundance of proteins or metabolites in the five cultivars in this study. Berry physiology was also unaffected by water stress, which indicated that the stress was mild. Matthews et al. have shown that mild water deficit does not significantly affect levels of soluble sugars, titratable acidity or berry diameter [102–104]. These grapes, however, did produce wines with significantly different flavor and aroma profiles [26]. In contrast, more severe water deficit causes significant reductions in berry diameter in Cabernet Sauvignon [31] and Chardonnay [27] and significantly alters metabolite composition and abundance. The lack of significant differences observed in the present study was possibly related to the mild water deficit, thus inducing only small differences in metabolite abundance. With a higher number of replications and lower CV for the samples, statistically significant changes in metabolite abundance in response to water deficit may have been detected.

Another explanation for only a few molecules with significant differences may be attributable partly to the single sampling time point at maturity. Dai et al. [105] surveyed a number of central metabolic signatures from whole berry samples displaying developmental specificity, with large abundance changes primarily occurring shortly before, through, and shortly after veraison. This argument is further supported by a fruit development experiment comparing Cabernet Sauvignon and Shiraz berry skins [45], which showed similar developmental trends in both central and secondary metabolites where large metabolic changes occurred early in development rather than at near-maturity. Additionally, the mild water deficit very likely caused subtle Omics changes that made it difficult to detect common responses with this level of replication.

Post-veraison, the berry undergoes rapid cellar expansion and increases in soluble sugars for a time, but as development continues, progressively towards senescence, the berry undergoes withering or dehydration. Perhaps the poor detection of treatment related effects was simply due to both treatments having experienced a degree of water deficit-related stress, although no visible withering or shrivel was observed. The high observed abundance of peroxiredoxin proteins across cultivars is consistent with the fact that they are known to be elevated in vines exposed to water deficit [36], although other environmental stress factors such as high light or UV intensity could also influence protein abundance. In an extreme example, Corvina berries undergo a withering process in the process of making the famous Ripasso and Amarone wines [79]. As a result of the mild water deficit used in this study, cultivar effects were the dominant differentiating factor in metabolic content.

## Conclusions

In summary, this study provides a rare and powerful glimpse into the molecular underpinnings of grape. It compares the skin of the berries of five cultivars of grapevines at maturity, a tissue that is a source of texture, color, flavor and aroma for grapes and wines. An enormous amount of effort and money went into collecting these data, but there was a much greater effort expended for the complex data analyses in this study. Few plant studies have collected such a large amount of data from five molecular platforms from identical tissue samples coming from a very similar environment (vineyard). The comparisons showed that the platforms were concordant with each other, that each variety can be distinguished from each other in a similar way with each of the platforms. Yet each platform was reproducible, providing a unique view, showing unique differences at each molecular level, and revealing some of the complexity of these biochemical pathways.

The phenotypic variation in the cultivars resulted in unique and large differences in abundance in many of the most common classes of proteins and metabolites measured in berry skins. Analysis of functional categories showed that metabolism was very active and there were substantial responses to abiotic and chemical stimuli in the berry skin. In this study, only transcript analyses were sensitive enough to detect significantly induced changes from the moderate water deficit treatment. Overall, transcript abundance was poorly correlated with protein abundance. Omic analyses elucidated cultivar differences in phenylpropanoid biosynthesis and amino acid metabolism that influence winemaking, including color, astringency and yeast assimilable nitrogen levels. In addition, this study showed that there were significant differences in the classes of pathogenesis proteins in the berry skins of each cultivar in the absence of pathogenic pressures.

The models presented here are simple and crude. One of the goals of the systems biology approach (Omics) is to construct models and make predictions. This study represents only the first steps in the path of achieving such goals. The integration of the data here and the models constructed are just the beginning. These simple models showed again that biochemical pathways are complex and cultivars can vary significantly in simple primary metabolic pathways, such as amino acid metabolism, as well as more complex secondary metabolic pathways, such as phenylpropanoid metabolism. To be predictive, more data will need to be collected over time to better estimate molecular activities and transport. Nonetheless, this study is valuable, depositing a large amount of information into public data repositories that can be used to build and create future molecular annotation and models. The data presented here can be utilized and explored for years to come.

## Methods

### Plant material and experimental conditions

Berries from five grapevine (*Vitis vinifera* L.) cultivars, Cabernet Sauvignon, Merlot, Pinot Noir, Chardonnay and Semillon, were harvested during the fall of 2011 from the University of Nevada, Reno experimental vineyards (Additional file 1). The North Vineyard was divided in half and separated into 15 rows (5-well watered; 10-drought stressed), with Chardonnay on the northern half and Cabernet Sauvignon on the southern half. Each row in the North Vineyard maintained 23 vines of each cultivar. The South Vineyard was divided into six blocks (A-F). Each block contained four rows divided into thirds, with 15 vines of a given cultivar in each third. Merlot, Pinot Noir & Semillon vines were grown in each block. Blocks A, C & D were well watered, and blocks B, E & F were treated with water deficit. Rows in each of the experimental vineyards were planted in a north to south orientation, to achieve nearly maximal daily sunlight exposure. Following fruit set in early July 2011, leaves were removed near the clusters on the east-facing side of vines in both vineyards to increase fruit exposure to light and air circulation. Vines were drip irrigated with 8 l h^−1^ emitters and grown under well-watered or water deficit conditions post-fruit set (Additional files 1 & 2). Mid-day stem water potentials were measured weekly with a pressure chamber (3005 Plant Water Status Console, Soil Moisture Corp., Goleta, CA, USA), as in [31], on fully mature leaves to assess plant water status throughout the growing season [106, 107]; stem water potential measurements were averaged across cultivars, because no significant differences in stem water potentials amongst the cultivars could be detected. Following weekly measurements, water was either applied or withheld in an effort to maintain a mild water deficit treatment at ~ −0.8 MPa and −0.6 MPa for control vines. Titratable acidity (TA) and °Brix (total soluble solids) were assayed from juice crushed from a minimum of two whole berry clusters collected from different vines. The TA (g l^−1^) measurements were performed with an automatic titrator (HI 84102, Hanna Instruments, Woonsocket, RI, USA). The automatic titrator was standardized daily with tartaric acid (6.4 g l^−1^), with 0.5 N NaOH utilized as a titrant to an endpoint of a pH of 8.2 for both standard and juice measurements. °Brix was measured with a digital refractometer (HI 96811, Hanna Instruments, Woonsocket, RI, USA) that was calibrated with deionized water before each measurement. Daily precipitation, Penman evapotranspiration and temperature measurements (Fig. 1) from the experimental vineyards were collected from the Desert Research Institute’s (DRI) Western Regional Climate Center [108]. DRI calculates evapotranspiration using the 1982 Kimberly-Penman equation [109]. Berry diameter measurements were taken weekly with a digital caliper (General Ultratech No. 147, New York, NY, USA), beginning after fruit set until the week of cultivar harvest. Berry diameter measurements consisted of measuring 15 randomly selected berries per cluster from the same four labeled clusters (technical replicates) on a single vine (experimental replicate). Three experimental replicates per cultivar and treatment were used to compute diameter means. Six experimental replicates, comprised of ≥ 2 whole berry clusters were harvested in early to late October 2011 (see Additional file 1 for details). Sampling dates for berry skin material varied between cultivars in order to achieve similar °Brix and TA concentrations in berries, but WW and WD treatments were gathered on the same day (Fig. 1; Table 2). To avoid edge effects, berry clusters were harvested from vines away from the ends of the trellised rows. All six sampled experimental replicates come from six different individual vines at different locations within the vineyard and were utilized for metabolomic extractions and analysis. From the six experimental replicates sampled, five experimental replicates were randomly selected for microarrays. Of the five experimental replicates used for microarrays, three experimental replicates were randomly selected for proteomic and RNAseq analysis. Thus, proteomic and RNAseq analyses utilized the same sampled experimental replicates. Berry skin tissue for all analyses was separated from the seeds and pulp prior to being flash frozen with liquid nitrogen and finely ground using a RETCH-mill (Retsch MM301, Newtown, PA, USA) with pre-chilled steel holders and grinding beads.

### Protein extraction and LC-MS/MS analysis

Proteins were extracted from the frozen, finely-ground skin samples using a modified phenol-based extraction protocol [34, 35]. Isolated protein pellets were prepared similarly to Cramer et al. [36] for label-free shotgun proteomics by Lys-C- and trypsin-digestion using a modified method of the Filter-Aided Sample Preparation (FASP) methods [110, 111], using trifluorethanol (TFE/FASP) [35]. LC-MS/MS spectra were acquired from three experimental replicates per treatment by a sample-optimized gas phase fractionation (GPF) method on a LTQ Velos Pro mass spectrometer (Thermo). Chromatography was performed on an Easy-nLC II (Thermo) at 40 ° C, using a 0.1 X 300 mm Magic 3 μm, 200 Å C18AQ column (Michrom Bioresources, Auburn, CA, USA) interfaced with the mass spectrometer by an Advance captive spray source (Michrom Bioresources). Samples were analyzed in three 220 min LC-MS/MS gas phase fractions run at 0.5 μL min^−1^. The m/z ranges of each gas phase was optimized empirically by analyzing a mixture of pooled samples from m/z 400–2000, then creating GPF fractions to approximate an even distribution of peptide observations among the three fractions.

A protein database was compiled from three sources: 1) all reviewed *V. vinifera* protein entries in UniProt, “Taxonomy:29760 AND reviewed:yes” (164 sequences); 2) *V. vinifera* proteins predicted by the International Grape Genome Program (29803 sequences); 3) mitochondrial proteins associated in UniProt (81 non-redundant sequences). Peptide to spectrum matching was performed with the X!Tandem algorithm running under the GPM Cyclone XE interface (www.thegpm.org, version 2011.12.01.1). Default ion trap parameters were used with the exceptions of MS error (+3, −1 Da), the inclusion of reversed sequences, and a protein expect value of −1. Approximately 50,000 spectra per sample were assigned to peptides. Protein identifications were filtered and protein and peptide FDRs were calculated, respectively, using reverse database searching. Each protein had to meet two criteria to be considered a valid identification. First, it must be present in all three biological replicates with a minimum of one spectral count in each replicate, of at least one variety at one condition; second, the sum of spectral counts was ≥ 6. Protein abundance was calculated as normalized spectral abundance factors (NSAF), using the Scrappy program, and a spectral fraction of 0.5 was added to all spectral counts to compensate for null values and therefore allow log transformation of data prior to subsequent statistical analyses [112].

### RNA extraction

Total RNA was extracted from ~250 mg of finely ground skin tissue using a modified CTAB extraction protocol based on [37–40] followed by an additional on-column DNase digestion using a Qiagen RNeasy Mini Kit (Qiagen, Valencia, CA, USA). RNA quality and quantity were assessed with a Nanodrop ND-1000 spectrophotometer (ThermoFisher Scientific, Waltham, MA, USA) and an Agilent 2100 Bioanalyzer and RNA LabChip assays (Agilent Technologies, Santa Clara, CA, USA).

### Microarray hybridization and data extraction

Ten μg of total RNA from each sample was used for hybridization onto a NimbleGen microarray 090818 Vitis exp HX12 (Roche, NimbleGen Inc., Madison, WI, USA), which contains probes targeted to 29,549 grapevine genes predicted from the V1 annotation of the 12x grapevine genome (https://urgi.versailles.inra.fr/Species/Vitis/Annotations). cDNA synthesis, labeling, hybridization, and washing steps were performed by MOgene (St. Louis, MO, USA) according to the NimbleGen Arrays User’s Guide (version 3.2). Data were processed, normalized and analyzed as in [41]. As in Cramer et al. [41], a note of caution should be held when examining the microarray data sets due to the likelihood of cross-hybridization of certain *Vitis* gene families with high similarity and are denoted in pink in Additional file 5.

### RNAseq library preparation and sequencing

For RNAseq, thirty 50 bp single-end, barcoded libraries were constructed and sequenced by the Neuroscience Genomics Core at the University of California Los Angeles (Los Angeles, CA, USA) using Illumina TruSeq RNA library prep kits (Illumina Inc., San Diego, CA, USA) according to the manufacture’s instructions. The libraries were pooled, multiplexed and run across eight lanes of four 1x50 flow-cells, using Illumina TruSeq chemistry (version 3.0) and a HiSeq2000 sequencer (Illumina Inc., San Diego, CA, USA). Due to multiplexing, individual experimental replicates were thus sequenced on each of the four flow-cells to reduce technical variation.

### Read quality and mapping pipeline

Quality check and filtering of fastq files was performed with the NGS QC Toolkit [113], prior to merging multiplexed replicate files. The TopHat2 splice alignment software (version 2.0.10) [114] in combination with the PN40024 *Vitis vinifera* reference genome and annotation (http://plants.ensembl.org/Vitis_vinifera/Info/Index) were used to align the quality filtered reads, with the --b2-very-sensitive option and --transcriptome-index option. Approximately 93 % of reads from all libraries were mapped. A count matrix of aligned reads was generated with Samtools [115] and HTSeq [43] from BAM alignment files, which outputs counts for each gene feature. Using the “union” mode, HTSeq discarded read counts if they were ambiguous, not assigned to any gene feature, or if the alignment was not unique.

### Data analysis

The ANOVA and most data analyses were conducted in R (3.1.2) [116]. RNAseq read count normalization and differential expression analysis were performed with edgeR (3.8.6) [44], counts from each aligned sample library (experimental replicate). An experimental design model was created accounting for cultivar (5 levels), treatment (2 levels) and the interaction between these two effects before fitting generalized linear models to estimate log-fold changes. Contrast coefficients for each factor were selected for significance testing. Moderated log-counts-per-million (Additional file 6) were computed with the cpm() function in edgeR for data visualization of RNAseq data.

### Gene set enrichment analysis

Functional analysis and enrichment of biological processes was determined with the BinGO (version 3.0.2) [47] application in Cytoscape (version 3.1.1) [48]. Multiple testing correction adjusted p-values using the Benjamini & Hochberg False Discovery Rate at a 0.05 threshold. Overrepresented GO terms were visualized with a treemap using REVIGO (http://revigo.irb.hr/) [49] and the treemap R package.

### GC and LC/MS metabolite analysis

Metabolite extraction was performed on aliquots of the same finely ground tissue samples utilized for protein extraction above and kept at −80 °C until further analysis. Briefly, skin samples were freeze dried in a lyophilizer (Labconco FreeZone 18, Kansas City, MS, USA) and extracted from 70 mg of frozen tissue with a pre-chilled methanol:chloroform:water (2.5:1:1 v/v), for parallel metabolite profiling (LC and GC/MS) using a protocol described previously [45]. GC-MS samples were re-dissolved and derivatized as described previously [117]. An AS 3000 autosampler, a TRACE GC ULTRA gas chromatograph, and a DSQII quadrupole mass spectrometer (Thermo-Fisher Ltd.) comprised the GC-MS system, with system parameters identical to those described in [117, 118]. LC-MS analysis was performed on an UPLC-QTOF-MS system equipped with an ESI interface (Waters Q-TOF XEVO, Waters MS Technologies, Manchester, UK), in negative and positive ion mode. An Acquity UPLC BEH C18 column (100 mm x 2.1 mm, 1.7 μm) was used for chromatographic separation. The MS and solvent gradient program conditions were set as described previously [117].

### Metabolite data processing

GC-MS spectral searching against the RI libraries from the Max-Planck Institute for Plant Physiology in Golm Germany (http://csbdb.mpimp-golm.mpg.de/csbdb/gmd/msri/gmd_msri.html) was performed in the Xcalibur data software (version 2.0.7), with the National Institute of Standards and Technology (NIST, Gaithersburg, USA) algorithm. These metabolites were normalized by the total metabolites and corrected for the dilution factor as in [45]. LC-MS data acquisition and UPLC system control was performed with the MassLynxTM software (Waters; version 4.1) as described in [117]. The verification of metabolite identification was done as described in [45].

### Availability of supporting data

The mass spectrometry proteomics data have been deposited with the ProteomeXchange [119] Consortium via the PRIDE partner repository with the dataset identifier PXD001661 and 10.6019/PXD001661. The microarray data discussed in this publication have been deposited in NCBI’s Gene Expression Omnibus [120] and are accessible through GEO Series accession number GSE72421 (http://www.ncbi.nlm.nih.gov/geo/query/acc.cgi?acc=GSE72421). RNAseq data were deposited with the Sequence Read Archive database at NCBI with BioProject identifier PRJNA268857 [121].

## Additional files

Additional file 1:
**The University of Nevada, Reno’s Experimental Vineyard is located at 39°32’20.14” N by 119°48’22.00” W.** Grapevines were grown in two adjacent vineyards under independent irrigation controllers. Red lines designate rows of water-deficit treated vines and blue lines designate well-watered vines. The north vineyard was divided in half (dotted line), with Chardonnay (CD) grown in the upper half and Cabernet Sauvignon (CS) grown in the lower half. The south vineyard was divided into six blocks, each containing four rows, with three blocks allocated to either water-deficit or well-watered treated vines. The block and row locations for Merlot (ME), Pinot Noir (PN), and Semillon (SM) are indicated. The experimental replicate sampling scheme for each technology is depicted. Six experimental replicates for each treatment and cultivar were harvested. (PDF 570 kb) Additional file 2:
**Stem water potential measurements (MPa) for the North and South vineyards.** Water potential measurements were averaged across cultivars, Cabernet Sauvignon and Chardonnay in the North and Merlot, Pinot Noir and Semillon in the South. Symbols represent mean ± SE; n = 6 (North) and 9 (South). WW = well watered, WD = water deficit. (PDF 165 kb) Additional file 3:
**Annotation, protein spectral counts, Normalized Spectral Abundance Factor (NSAF) values and log2 transformed NSAF values for each replicate and protein identified, with ‘.count’, ‘.NSAF’, and ‘.NSAF.log2’ suffixes, respectively.** Mass weight (Mr) and amino acid (AA) length are approximated values. Cultivar and treatment abbreviations for experimental replicates: Cabernet Sauvignon (CS), Merlot (ME), Pinot Noir (PN), Chardonnay (CD), and Semillon (SM) grown under well-watered (W) and water deficit (D) conditions, n = 3. Other column headings refer to a combination of cultivar name, treatment, and replicate number. For example, CDD2, is Chardonnay, water deficit and replicate 2. (XLS 4096 kb) Additional file 4:
**ANOVA results for the quantifiable (1211) proteins (log2 NSAF) in five grape cultivars, with p-value (p) and adjusted p-value (adjp) prefixing each effect.** Cultivar and treatment abbreviations for experimental replicates: Cabernet Sauvignon (CS), Merlot (ME), Pinot Noir (PN), Chardonnay (CD), and Semillon (SM) grown under well-watered (W) and water deficit (D) conditions, n = 3. Other column headings refer to a combination of cultivar name, treatment, and replicate number. (XLS 1032 kb) Additional file 5:
**Annotation, transcript abundance values, and ANOVA results of all genes on the NimbleGen Whole-Genome microarray measured in five grape cultivars.** Red highlighted rows identify the possibility of cross-hybridization of probes with other genes from Cramer et al. [41]. Cultivar and treatment abbreviations for experimental replicates: Cabernet Sauvignon (CS), Merlot (ME), Pinot Noir (PN), Chardonnay (CD), and Semillon (SM) grown under well-watered (W) and water deficit (D) conditions, n = 5. The probe.cross.hybridization column refers to the number of probes for that transcript that have the potential to cross-hybridize with other probes. Other column headings refer to a combination of cultivar name, treatment, and replicate number. (XLS 33741 kb) Additional file 6:
**Annotation, read counts, transcript normalized log2 counts per million (CPM) values, and edgeR statistical results of all genes with unique counts assigned from Illumina RNAseq, with ‘.count’ and ‘.log2CPM’ suffixes respectively.** Cultivar and treatment abbreviations for experimental replicates: Cabernet Sauvignon (CS), Merlot (ME), Pinot Noir (PN), Chardonnay (CD), and Semillon (SM) grown under well-watered (W) and water deficit (D) conditions, n = 3. Other column headings refer to a combination of cultivar name, treatment, and replicate number. (XLS 25195 kb) Additional file 7:
**Mean relative abundance values, M/Z, and results from the ANOVA for all primary and secondary metabolomic details for all metabolites (67) analyzed by GC-MS and (42) analyzed by LC-MS in five grape cultivars.** Cultivar and treatment abbreviations for experimental replicates: Cabernet Sauvignon (CS), Merlot (ME), Pinot Noir (PN), Chardonnay (CD), and Semillon (SM) grown under well-watered (W) and water deficit (D) conditions, n = 6. (XLS 76 kb) Additional file 8:
**BinGO results for overrepresented GO biological process functional categories for all quantifiable proteins (1211).** The proteins are identified by their UniProtKB accession name. (XLS 303 kb) Additional file 9:
**Correlations of protein and transcript abundance.** Protein data are log2 NSAF values, n = 3, RNAseq data are log2 normalized counts per million (CPM), n = 3, and microarray data are log2 RMA values, n = 5. Relationships of proteins with either RNAseq (CPM) or microarray (RMA) are indicated. (XLS 370 kb) Additional file 10:
**The effect of water deficit upon the relative metabolic content of five anthocyanidins and their glycosylated, acetylated and coumaroylated moieties within the red cultivars.** All metabolites were significant at the Cultivar level except malvidin 3-O-(6-p-coumaroyl)glucoside and petunidin 3-O-(6-acetyl)glucoside. Cultivar and treatment abbreviations for experimental replicates: Cabernet Sauvignon (CS), Merlot (ME) and Pinot Noir (PN), grown under well-watered (W) and water deficit (D) conditions. Error bars represent mean ± SE, n = 6. (PDF 13 kb)
