# Supplementary material for: Five omic technologies are concordant in differentiating the biochemical characteristics of the berries of five grapevine (Vitis vinifera L.) cultivars
Source: BMC Genomics. 2015 Nov 16;16:946. doi: 10.1186/s12864-015-2115-y (PMC4647476; doi:10.1186/s12864-015-2115-y)

- Vineyard is located at high elevation (1372 m) in a very dry climate
  - Split-rows in North (a)
  - Randomized-block design in South (b)
- 13 cultivars grown in vineyards
  - 7 red and 6 white
- Different rows under different irrigation controls
  - Stem water potentials of -0.6 MPa for control vines and -0.8 MPa for water deficit

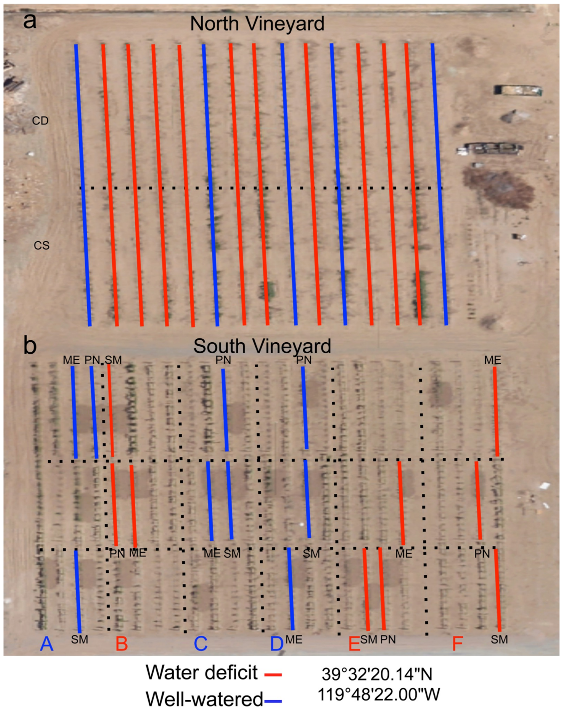

6x Experimental replicates sampled, ≥ 2 whole berry clusters

Metabolomics: 6x experimental replicates

Microarrays: 5x experimental replicates, randomly sampled from 6x experimental replicates from metabolomics

Proteomics & RNAseq: 3x experimental replicates, randomly sampled from 5x experimental replicates from microarrays

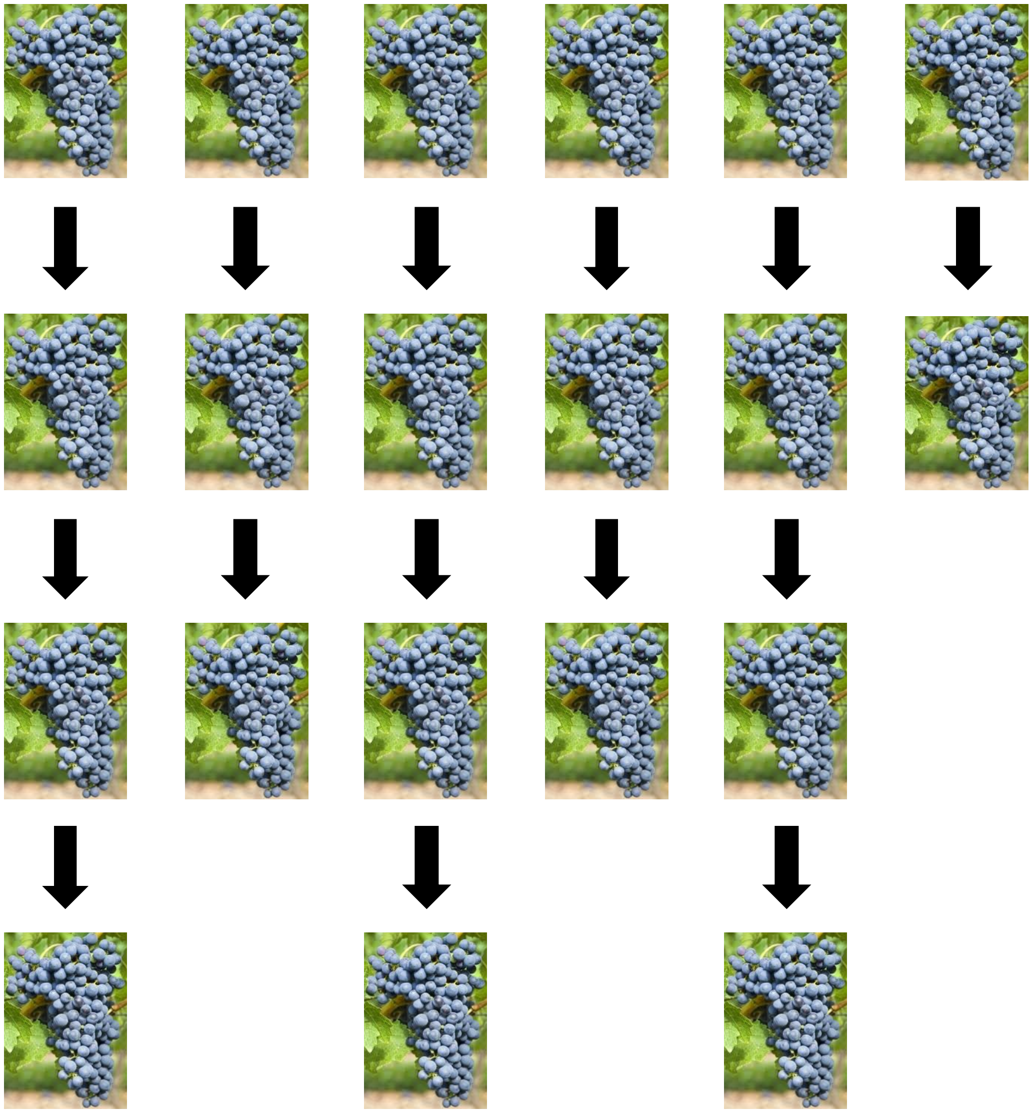

Supplement: Additional file 1: — The University of Nevada, Reno’s Experimental Vineyard is located at 39°32’20.14” N by 119°48’22.00” W. Grapevines were grown in two adjacent vineyards under independent irrigation controllers. Red lines designate rows of water-deficit treated vines and blue lines designate well-watered vines. The north vineyard was divided in half (dotted line), with Chardonnay (CD) grown in the upper half and Cabernet Sauvignon (CS) grown in the lower half. The south vineyard was divided into six blocks, each containing four rows, with three blocks allocated to either water-deficit or well-watered treated vines. The block and row locations for Merlot (ME), Pinot Noir (PN), and Semillon (SM) are indicated. The experimental replicate sampling scheme for each technology is depicted. Six experimental replicates for each treatment and cultivar were harvested. (PDF 570 kb) [file 12864_2015_2115_MOESM1_ESM.pdf]
