# Supplementary figures and images for: Five omic technologies are concordant in differentiating the biochemical characteristics of the berries of five grapevine (Vitis vinifera L.) cultivars
Source: BMC Genomics. 2015 Nov 16;16:946. doi: 10.1186/s12864-015-2115-y (PMC4647476; doi:10.1186/s12864-015-2115-y)

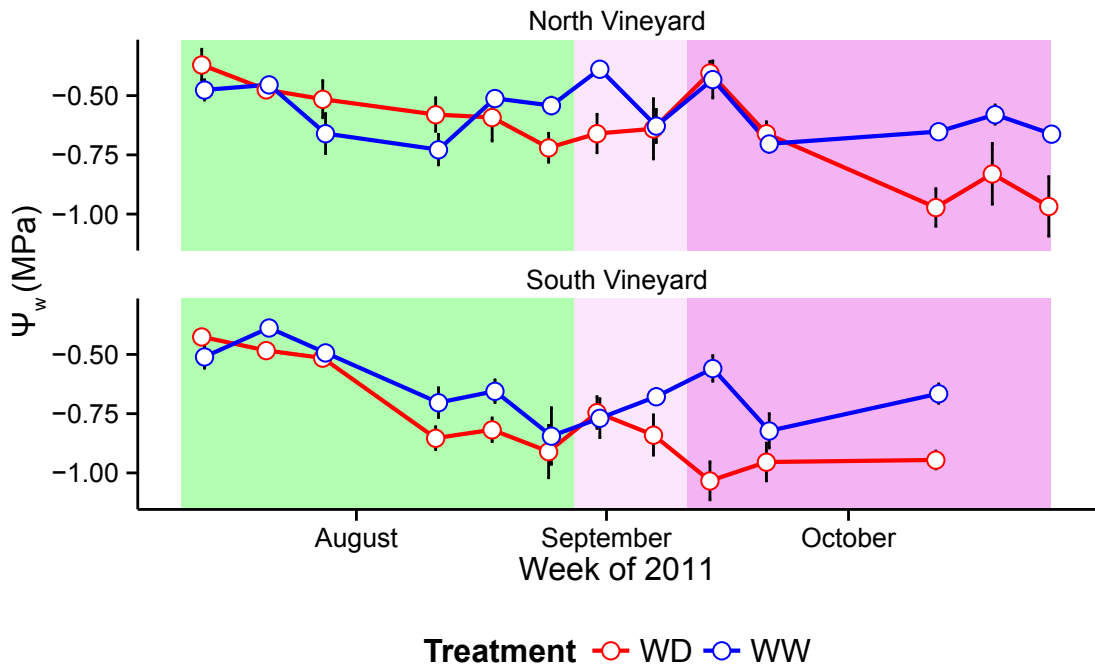

Supplement: Additional file 2: — Stem water potential measurements (MPa) for the North and South vineyards. Water potential measurements were averaged across cultivars, Cabernet Sauvignon and Chardonnay in the North and Merlot, Pinot Noir and Semillon in the South. Symbols represent mean ± SE; n = 6 (North) and 9 (South). WW = well watered, WD = water deficit. (PDF 165 kb) [file 12864_2015_2115_MOESM2_ESM.pdf]

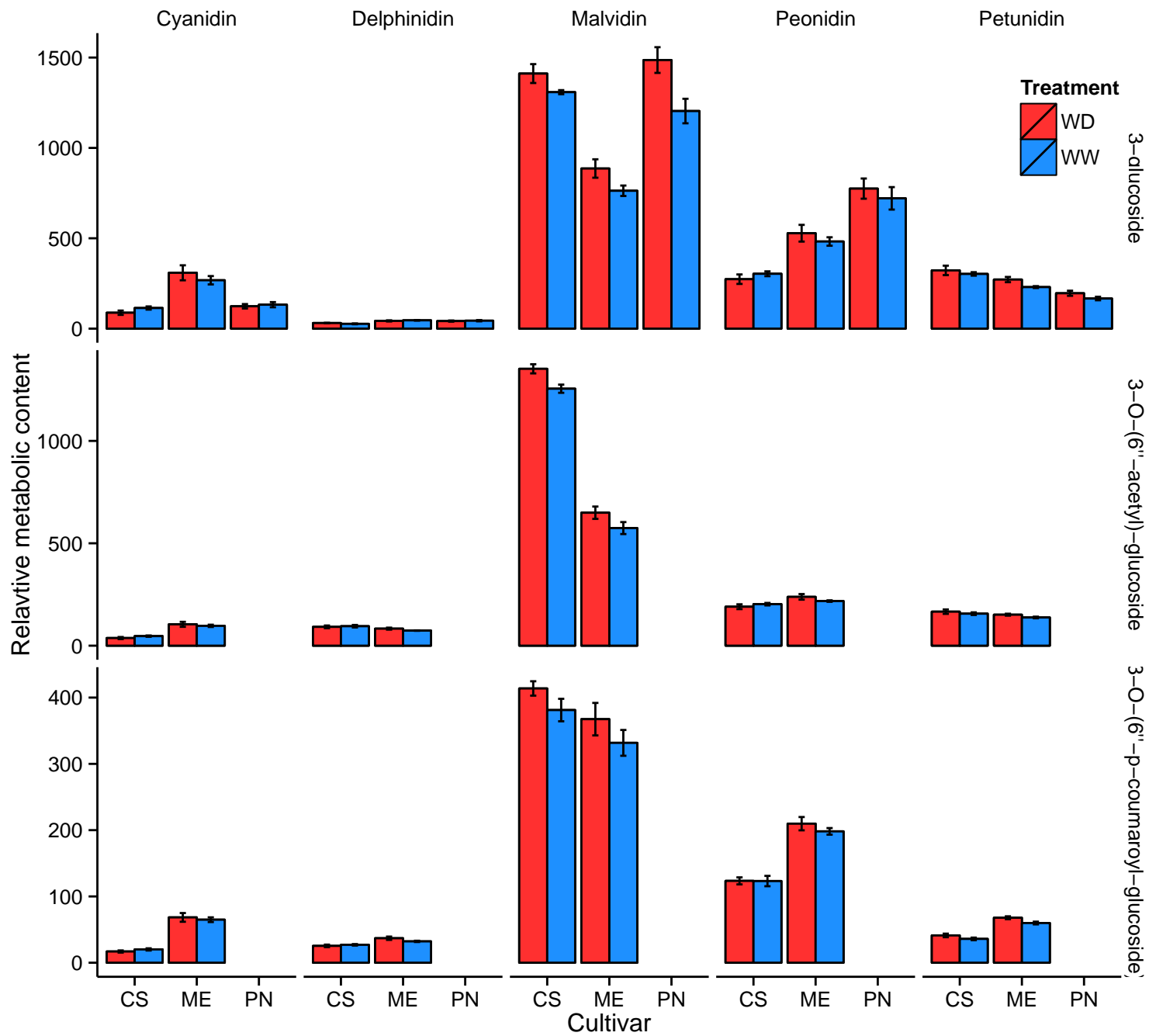

Supplement: Additional file 10: — The effect of water deficit upon the relative metabolic content of five anthocyanidins and their glycosylated, acetylated and coumaroylated moieties within the red cultivars. All metabolites were significant at the Cultivar level except malvidin 3-O-(6-p-coumaroyl)glucoside and petunidin 3-O-(6-acetyl)glucoside. Cultivar and treatment abbreviations for experimental replicates: Cabernet Sauvignon (CS), Merlot (ME) and Pinot Noir (PN), grown under well-watered (W) and water deficit (D) conditions. Error bars represent mean ± SE, n = 6. (PDF 13 kb) [file 12864_2015_2115_MOESM10_ESM.pdf]
